# Biomolecular Interaction Prediction: The Era of AI

**DOI:** 10.1002/advs.202509501

**Published:** 2025-07-16

**Authors:** Haoping Wang, Xiangjie Meng, Yang Zhang

**Affiliations:** ^1^ School of Biomedical Engineering Harbin Institute of Technology (Shenzhen) Shenzhen Guangdong 518055 China; ^2^ School of Pharmaceutical Science and Technology Tianjin University Tianjin 300072 China

**Keywords:** biomolecular interaction, deep learning, nucleic acid, protein, small molecule

## Abstract

Predicting biomolecular interactions is a crucial task in drug discovery and molecular biology. Deep learning, with its ability to learn complex patterns from large datasets, has shown promising results in predicting biomolecular interactions. In this review, a comprehensive and accessible overview of deep learning algorithms is aimed to provide that can enhance the prediction of biomolecular interactions using various features, including sequence data, structural information, and functional annotations. The datasets and models for predicting biomolecular interactions using deep learning are summarized. These deep learning models are developed for a wide range of target molecules, including proteins, nucleic acids, and small molecules, thus reducing the time and cost of screening compounds with high binding affinity to a given target. Furthermore, deep learning can also aid in understanding the mechanisms of biomolecular interactions by identifying key residues involved in the interaction, and help in predicting the side effects of drugs by identifying potential off‐target interactions. In conclusion, deep learning has the potential to revolutionize drug discovery and improve understanding of molecular biology by providing accurate and efficient prediction in biomolecular interactions.

## Graphical abstract

This review offers a thorough examination of recent progress in deep learning for predicting biomolecular interactions, including those involving proteins, nucleic acids, and small molecules. It covers data processing strategies, representative model architectures, and evaluation metrics, while highlighting current methodological limitations.

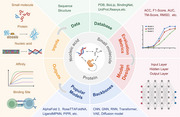

## 1 How AI Illuminates Biomolecular Interaction Prediction

Artificial intelligence (AI) has rapidly transformed many scientific fields, offering innovative solutions to complex challenges once constrained by human or computational limitations.^[^ ^1^, ^2^ ^]^ Biomolecular interaction prediction is one area that stands to benefit significantly from AI advancements. This field is critical in drug discovery, molecular biology, and genomics research.^[^ ^3^, ^4^, ^5^, ^6^, ^7^, ^8^ ^]^ AI and deep learning methods have made significant advancements in biomolecular interaction prediction, providing substantial improvements over traditional computational techniques such as molecular docking and statistical models in recent years. Deep learning has revolutionized biomolecular interaction prediction by developing models capable of learning from large and diverse datasets, identifying hidden patterns, and delivering more accurate predictions.^[^ ^9^, ^10^, ^11^ ^]^ These models integrate data from various sources, such as sequence data, structural information, and biological annotations, thereby offering insights that are not easily attainable through traditional computational methods. Beyond predicting interactions, AI can provide deeper insights into biological mechanisms with improved identification of critical binding sites or residues involved in the interaction.^[^ ^12^ ^]^ These advancements are essential for managing the complexity and large scale of interaction data, providing more accurate predictions, speeding up drug discovery, and enhancing our comprehension of biological systems.

**Figure 1:**
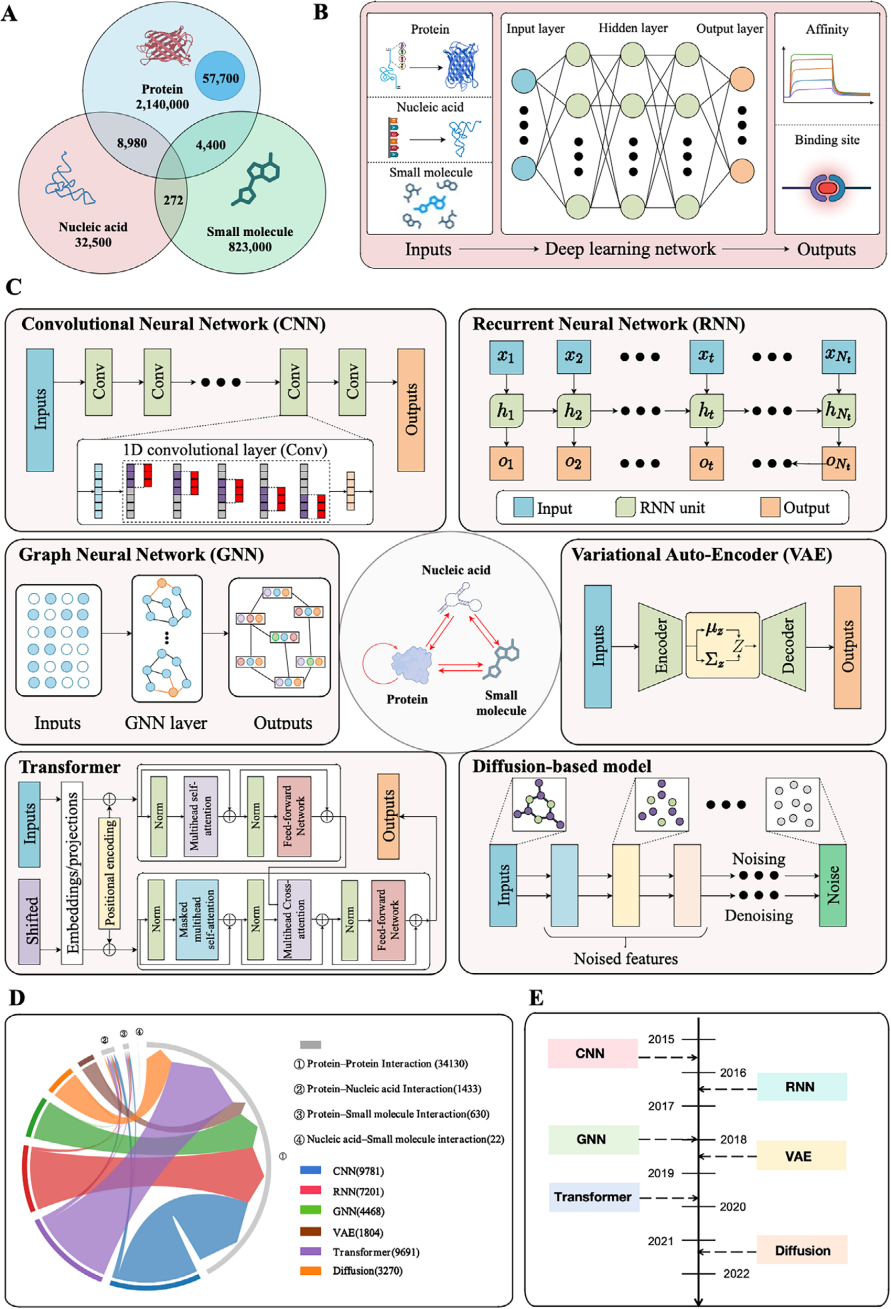
Deep learning‐based frameworks for biomolecular interaction prediction. A) The Venn diagram shows the number of publications in Google Scholar from 2015 to 2025 on deep learning‐based biomolecular interaction studies. Most studies focus on proteins and their ligands, while studies involving nucleic acids and small molecules remain limited. B) Schematic workflow of deep learning‐based biomolecular interaction prediction. Typical models take biomolecular structures or sequences as input, process them through multiple hidden layers in a neural network, and output predictions  such as binding affinity or binding sites. C) Representative neural network architectures used for biomolecular interaction prediction. From top left to bottom right: Convolutional Neural Network (CNN) captures spatial features from biomolecular sequences via stacked convolutional layers. Recurrent Neural Network (RNN) models sequential dependencies, especially useful for temporal or ordered biological data. Graph Neural Network (GNN) encodes molecular structures as graphs, enabling the modeling of interactions at the atomic or residue level. Variational Autoencoder (VAE) learns latent representations of biomolecular features through encoding and decoding processes, useful for generation and design tasks. Transformer networks, which leveraging attention mechanisms and positional embeddings, are effective for modeling long‐range dependencies in sequences and structures. Diffusion‐based models adopt a generative paradigm involving noise addition and removal steps, capturing complex molecular conformations and enabling structure generation. D) The chord diagram illustrates the relationships between different deep learning model types and biomolecular interaction tasks based on publication counts retrieved from Google Scholar between 2015 and 2025. Each segment represents a model (left half) or interaction type (right half), witharc widths indicating the number of studies involving a given model‐task combination. Interaction tasks cover protein–protein interaction, protein–nucleic acid interaction, protein–small molecule interaction, and nucleic acid–small molecule interaction. The figure highlights that most studies are concentrated in protein–protein interactions, particularly using Transformer and CNN architectures, while nucleic acid‐related interactions remain underrepresented. E) Timeline of major developments in neural network applications for biomolecular interaction research from 2015 to 2025. CNN and RNN were early pioneers, followed by GNN and VAE for molecular structure modeling and generation. From 2020 onward, Transformer‐based models and Diffusion models significantly advanced the field by enabling high‐resolution structural prediction and interaction modeling.

The current landscape of deep learning research in the prediction of biomolecular interaction has predominantly focused on proteins and their interacting partners, including the study of protein‐protein interactions (PPIs), protein‐nucleic acid interactions, and protein‐small molecule interactions.^[^ ^13^ ^]^ These areas have garnered significant attention due to the critical roles of proteins in biological processes and their involvement in various diseases. Deep learning models are highly effective in predicting protein structures, enhancing our understanding of their binding mechanisms, and identifying potential drug candidates targeting proteins.^[^ ^14^ ^]^ The nucleic acid‐related interactions are crucial for bioprocesses such as transcription, translation, and post‐translational modifications. Based on a survey of papers indexed in Google Scholar from 2015 to 2025, it is evident that nucleic acid‐related studies are relatively scarce compared to those focusing on proteins (**Figure** **1**A). This is likely due to the limited availability of nucleic acid data and the structural complexity of nucleic acids.^[^ ^15^ ^]^ Similarly, studies concerning small‐molecule interactions have remained notably underrepresented. Small molecules act as versatile players in biomolecular interactions, often functioning as ligands that bind to proteins, nucleic acids, or other small molecules to modulate their function.^[^ ^16^ ^]^ These interactions are essential to drug discovery and the understanding of disease mechanisms. Regardless of the specific AI methods and data employed, all those studies adhere to a comparable protocol. The general workflow of deep learning methodologies in investigating biomolecular interactions encompasses inputs, AI models, and outputs (Figure 1B). Sequence and structure information about proteins, nucleic acids, and small molecules is encoded and input into deep learning models, which are designed and trained based on specific task objectives and model architectures (as detailed in Figure 1C and discussed later in the text). The models then generate predictive outputs such as binding affinity, interaction sites, or other relevant molecular properties. Over the recent decade (2015–2025), research has predominantly centered on protein‐protein interactions, with Transformer and CNN architectures emerging as prominent methodologies (Figure 1D). This is because CNN, as a relatively older backbone, has been widely used in molecular interaction research since around 2015. In contrast, the Transformer architecture gained popularity more recently, around 2020, exemplified by renowned models like AlphaFold3^[^ ^17^ ^]^ (Figure 1E).

By utilizing large datasets and advanced computational models, AI can design and predict biomolecular interactions of proteins, nucleic acids, and small molecules with high accuracy and efficiency. This not only streamlines drug discovery but also opens new avenues for exploring fundamental biological processes, thereby improving our understanding of biomolecular mechanisms and systems.

## 2 High‐Quality Data: The Foundation for AI‐Driven Biomolecular Interaction Prediction

The application of deep learning in studying biomolecular interactions generally involves four primary stages: data preprocessing, model construction, result prediction and validation, and downstream analysis applications.^[^ ^14^, ^18^, ^19^, ^20^ ^]^ In the data preprocessing stage, researchers obtain biomolecular sequence, structure, and interaction data from experimental measurements or databases and optimize the input data through feature extraction, standardization, and data augmentation to enhance the model's learning capacity and generalization.^[^ ^18^ ^]^ Subsequently, the model construction stage involves selecting and designing deep neural networks, including convolutional neural networks (CNNs) for local feature extraction, recurrent neural networks (RNNs) for sequence dependency modeling, Transformer architectures for global information learning, and graph neural networks (GNNs) for molecular topology relationships.^[^ ^19^, ^21^, ^22^ ^]^ During training, the model optimizes its parameters through supervised or self‐supervised learning strategies to accurately identify biomolecular interaction patterns. In the prediction and validation stage, the trained model is used to make predictions on new data, and its performance is assessed using cross‐validation, evaluation with an independent test set, and ablation experiments to ensure the reliability of the results.^[^ ^23^ ^]^ Additionally, computational simulations or experimental data can be incorporated to validate biological plausibility, further enhancing the model's credibility.^[^ ^24^ ^]^ Finally, in the downstream application stage, researchers use predictions from deep learning models to enhance biological studies, such as helping in target discovery, optimizing molecular design, and investigating functional mechanisms, thereby supporting broader research in life sciences.^[^ ^25^, ^26^ ^]^

In the study of molecular interactions using deep learning, the selection and processing of data types are essential, as the quality and representation of data directly influence model performance and predictive capabilities. The data involved in molecular interaction research primarily include sequence data and structural data, which provide information on molecular composition and spatial arrangement, respectively.^[^ ^27^, ^28^ ^]^ These data types serve as the foundation for deep learning approaches in modeling biomolecular interactions.

Sequence data serve as the most fundamental and widely used format in molecular interaction studies. Due to the inherent linear nature of sequence data, researchers often adopt methods from natural language processing (NLP), treating protein or RNA sequences as “sentences” composed of amino acids or nucleotides and applying models such as RNNs, long short‐term memory networks (LSTMs), or transformers for feature extraction.^[^ ^29^, ^30^, ^31^, ^32^ ^]^ For example, protein language models (e.g., ESM, ProtBERT), which have recently gained prominence, leverage self‐supervised learning to pretrain on large‐scale biological sequence datasets. This process enables them to capture generalizable features that can be further fine‐tuned for predicting molecular interactions, functions, and structures.^[^ ^20^, ^33^ ^]^ However, relying exclusively on sequence data may not fully capture the physicochemical properties that govern molecular interactions, particularly those related to protein folding and spatial conformation. Thus, while sequence data play a critical role in molecular interaction studies, they are often supplemented by structural information to enhance predictive abilities and accuracy.^[^ ^34^ ^]^

In contrast, structural data provide 3D spatial information about molecules, including atomic coordinates, bond lengths, bond angles, and secondary and tertiary structural elements of proteins.^[^ ^35^ ^]^ Structural data primarily derive from experimental techniques such as X‐ray crystallography, nuclear magnetic resonance (NMR) spectroscopy, and cryo‐electron microscopy (Cryo‐EM), as well as computational predictions using homology modeling and deep learning‐based methods like AlphaFold.^[^ ^14^, ^36^, ^37^, ^38^, ^39^, ^40^ ^]^ Various representation methods exist for structural data, with graph‐based representations being the most common. In this approach, molecules are modeled as graphs where nodes correspond to atoms or amino acids and edges represent chemical bonds or spatial proximity relationships. This graph representation is particularly well‐suited for graph neural networks, enabling models to exploit molecular topology to learn more informative features.^[^ ^41^ ^]^ Additionally, structural data can be represented using 3D voxel grids, point clouds, or topological data analysis, allowing deep learning models to process spatial features more effectively.^[^ ^42^ ^]^ For instance, AlphaFold has shown remarkable success in predicting protein structures by integrating evolutionary information and physical constraints, resulting in high‐quality structural data that significantly advances molecular interaction research.^[^ ^14^ ^]^ However, acquiring structural data remains expensive, and challenges such as limitations in resolution and difficulties in capturing dynamic conformational changes persist.^[^ ^17^ ^]^ Thus, optimizing the use of structural data and integrating it with sequence data to enhance predictive capabilities remains an active research area.

Before inputting biomolecular data into deep learning models, data preprocessing is a crucial step that directly impacts model performance and prediction accuracy.^[^ ^43^ ^]^ Given the diversity and complexity of molecular data, proper preprocessing not only reduces noise and redundancy but also extracts task‐relevant features, enabling models to learn and generalize more effectively. When handling sequence data, the first step is to standardize the representation of protein, RNA, or small‐molecules sequences.^[^ ^44^ ^]^ Typically, amino acids or nucleotides are mapped to numerical representations, such as one‐hot encoding, embedding vectors, or representations derived from pretrained language models.^[^ ^45^ ^]^ Since sequence lengths vary, padding or truncation is often applied to ensure a uniform input size, which is crucial for efficient batch processing during model training.^[^ ^46^ ^]^ For structural data, preprocessing is inherently more complex due to the non‐Euclidean nature of 3D molecular structures. First, the atomic coordinates of proteins, RNAs, or small molecules must be extracted from experimental databases (e.g., PDB) or computationally predicted structures.^[^ ^47^ ^]^ Redundant structures, such as solvent molecules or crystallographic artifacts, must be removed. To enable deep learning models to effectively utilize 3D information, molecular structures are often represented as graphs, where nodes correspond to atoms or amino acid residues, and edges represent chemical bonds or spatial interactions. This graph‐based approach requires constructing adjacency matrices, normalizing graph structures, and applying GNNs, such as graph convolutional networks (GCNs) or graph attention networks (GATs), to extract meaningful features.^[^ ^48^, ^49^ ^]^ Another common approach is voxelization, which transforms molecular structures into 3D grids suitable for processing with 3D convolutional neural networks (3D‐CNNs).^[^ ^50^, ^51^, ^52^ ^]^ Alternatively, point cloud representations can be employed, where models like PointNet extract structural features directly from collections of atomic coordinates.^[^ ^53^, ^54^ ^]^ Since biomolecular structures frequently demonstrate conformational flexibility, molecular dynamics simulations can generate ensembles of conformations, enabling models to integrate structural dynamics into their predictions.

Over the past decade, deep learning has shown remarkable capabilities in studying protein and nucleic acid evolution, especially in generating homologous sequences from existing genetic data.^[^ ^55^ ^]^ Traditional approaches to evolution, such as alignment‐based methods and phylogenetic tree reconstruction, have been used for a long time to infer evolutionary relationships among sequences.^[^ ^56^, ^57^ ^]^ However, these methods often face limitations when dealing with highly variable sequences or those with low sequence similarity. With the advancement of deep learning technologies, generative models have emerged as powerful tools in nucleic acid sequence evolution, allowing researchers to generate novel functional homologous sequences and predict their structural and functional properties based on existing datasets. Generative models, including Variational Autoencoders (VAEs) (Figure 1C) and Generative Adversarial Networks (GANs), have been used to learn and generate nucleic acid sequences.^[^ ^58^, ^59^ ^]^ In addition to generative models, the Transformer architecture has increasingly been used for predicting sequence variations and evolutionary pathways.^[^ ^60^ ^]^ Compared to traditional alignment‐based methods, deep learning‐based sequence generation techniques excel at capturing long‐range dependencies within sequences and optimizing their adaptive potential in a data‐driven way. Moreover, diffusion models have recently gained traction in the field of protein and RNA sequence generation.^[^ ^61^ ^]^ These models simulate the dynamic processes of mutation and selection in biological evolution, gradually refining nucleic acid sequences toward specific evolutionary goals, such as improving binding affinity to target proteins or optimizing mRNA stability. In summary, sequence generation techniques are revolutionizing biomolecular interaction prediction by addressing data scarcity, improving model generalizability, and enabling the design of molecules with tailored properties. As generative AI advances, it will play an increasingly central role in drug discovery, ultimately accelerating our understanding of complex biomolecular interaction networks.

By prioritizing open access, standardization, and curation, public databases ensure that high‐quality data is readily available for AI research, fostering innovation and reproducibility in the field. To guide researchers in developing and systematically evaluating their AI models for biomolecular interaction prediction, this review summarizes key databases to ensure robust, reliable, and reproducible model development (**Table** **1**). Among them, PDB continues to be the gold standard for experimentally determined structures of proteins, nucleic acids, and complex assemblies.^[^ ^62^ ^]^ The PDBbind database gathers experimentally measured binding affinity data from all biomolecular complexes in the PDB.^[^ ^47^, ^63^ ^]^ The database's extensive data on binding affinities between proteins and ligands aids AI models in predicting how small molecules interact with biological targets, thereby facilitating the development of new therapeutic agents. BindingDB is another critical resource that focuses on protein‐ligand interactions.^[^ ^64^ ^]^ It contains detailed information on binding constants and enzyme inhibition constants, making it an invaluable tool for drug discovery. The database's extensive data on binding affinities between proteins and ligands helps AI models predict how small molecules interact with biological targets, facilitating the development of new therapeutic agents. DrugBank combines thorough drug data with comprehensive information on drug targets and pathways.^[^ ^65^ ^]^ This unique bioinformatics resource is particularly useful for developing models that predict drug‐target interactions. By providing insights into the pharmacological properties of various compounds, DrugBank aids in understanding drug mechanisms and identifying possible adverse effects. The Clinical Evaluation of Meta‐Analysis (ChEMBL) database offers information on bioactive molecules, including their biological activities, targets, and chemical properties and structures.^[^ ^66^ ^]^ ChEMBL's extensive data repository is crucial for training AI models to predict compound activity and interactions. This database facilitates the discovery of novel therapeutic agents by offering a wealth of information on known bioactive compounds and their effects. UniProt serves as a comprehensive resource that delivers detailed information on protein sequences and functional interactions.^[^ ^67^ ^]^ It includes a vast collection of annotated protein data, which is crucial for training AI models to predict protein functions and interactions. UniProt's integration with other databases and tools enhances its utility, making it an indispensable resource for AI‐based research. Reaxys provides comprehensive chemical reaction data, which can be integrated with deep learning models to predict reaction outcomes and molecular interations.^[^ ^68^ ^]^ By integrating and analyzing extensive chemical reaction data, Reaxys supports cheminformatics and assists in designing new compounds with desired binding properties. The ongoing updates and improvements to these databases ensure they remain at the forefront of AI research. By leveraging these evaluation metrics and public databases, researchers can rigorously evaluate and compare biomolecular interaction models, ensuring robustness and reliability in downstream applications like drug discovery or systems biology.

**Table 1:** Representative database in AI‐based biomolecular interaction prediction.

Database	Description	Main Features	Data Source	Data size	Key application	Website
PDB^[^ ^62^ ^]^	A repository of information about the 3D structures of large biological molecules.	Contains structural data on proteins, nucleic acids, and complex assemblies.	X‐ray crystallography, NMR spectroscopy, cryo‐EM	October 2024 195,210 protein, 13,680 Protein‐Nucleic Acid Complexes, 2513 DNA, and 1885 RNA data	Structure‐based drug discovery, molecular modeling.	https://www. rcsb.org/
PDBbind+^[^ ^47^, ^63^ ^]^	A repository containing binding affinity data for various biomolecular complexes	Binding affinity data (Kd, Ki, IC50), covering various biomolecular complexes; updated annually, data based on structures from PDB	PDB	August 2024 22,920 Protein‐ligand, 3,176 Protein‐Protein, 1,141 Protein‐nucleic acid, and 171 Nucleic acid‐ligand complexes	Protein‐ligand, protein‐protein, protein‐nucleic acid, nucleic acid‐ligand	https://www. pdbbind‐plus. org.cn/
BioLiP^[^ ^69^ ^]^	A curated repository for ligand‐protein binding interactions, providing high‐quality binding information extracted from PDB and the literature. It includes structural and functional annotations that aid in binding affinity research and analysis.	Includes a semi‐manual curation process to ensure data completeness and quality, suitable for binding affinity research	PDB data, literature, other databases	September 2024 460725 regular, 197122 metal, 39065 peptide, 45686 DNA, and 160800 RNA ligands	Proteins, DNA/RNA, peptides, metals, and regular ligands	https://zhanggroup. org/BioLiP/ index.cgi
BindingDB^[^ ^64^ ^]^	A public database that contains experimentally measured binding affinities between proteins and small molecules.	Focuses on binding affinity between proteins and drug‐like small molecules, provides tools for querying, analyzing, and downloading data.	Literature data	September 2024 Patents:7,349, Binding measurements: 1,142,149, Compounds: 559,074, Target proteins: 2,733, and Assays: 10,505	Protein‐small molecule interactions	https://www. bindingdb.org/ rwd/bind/index.jsp
BioGRID^[^ ^70^ ^]^	A comprehensive repository of protein‐protein, genetic, and chemical interaction data.	Integrates various interaction types, including protein‐protein, genetic, and chemical interactions; a comprehensive database suitable for mapping complex interaction networks	Experimental data, literature	October 2024 2,807,416 protein and genetic interactions, 31,144 chemical interactions, and 1,128,339 post‐translational modifications from major model organism species	Multiple organisms, including yeast, humans, and others	https://thebiogrid .org/
ChEMBL^[^ ^66^ ^]^	A comprehensive database of bioactive molecules with drug‐like properties.	Provides bioactivity data (binding, functional, ADMET) for over 1 million compounds and 5200 protein targets. Useful for drug discovery research, structure‐activity relationships (SARs), and predictive modeling. Accessible via web interface, downloadable datasets, and web services.	Literature: approved drugs and clinical candidates	October 2024 2,400,000 compounds, 1,600,000 assays,48,000 indications, 6,900 mechanisms, and 15,000 drugs and targets	Drug‐like compounds, protein targets, bioactivity data	https://www.ebi.ac. uk/chembl/
BindingNet^[^ ^71^ ^]^	A dataset designed to analyze protein‐ligand interactions, containing high‐quality modeled poses of compounds that are similar to crystal ligands found in PDBbind, as well as activity data from ChEMBL.	Contains high‐quality modeled poses for compounds similar to crystal ligands found in PDBbind, along with activity data from ChEMBL	PDBbind, ChEMBL	October 2024 802 targets, 69,186 complexes, 105,897 activities, 16,431 assays, and 5,907 pdbids	Protein‐ligand complexes	http://bindingnet. huanglab.org.cn/
DrugBank^[^ ^65^ ^]^	A comprehensive database that combines detailed drug data with drug‐target interactions.	Detailed chemical, pharmacological, and pharmaceutical data along with drug‐target interaction information,	Literature, scientific studies	October 2024 More than 15,000 drugs and 500,000 medications	Drug discovery and development, pharmacological research, providing insight into drug mechanisms and target interactions	https://go.drugbank.com/
UniProt^[^ ^67^ ^]^	A comprehensive resource for protein sequence and functional information.	High‐quality protein sequence and functional information, including protein functions and biological processes.	Literature, computational analysis	Oct 02 2024 572,214 Reviewed (Swiss‐Prot) and 248,266,673 Unreviewed (TrEMBL)	Protein function annotation, proteomics, and genomics research	https://www.uniprot.org/
Reaxys^[^ ^68^ ^]^	Reaxys is a chemistry database providing access to a vast collection of experimental data on chemical substances, reactions, and properties.	Vast collection of experimental data on chemical substances, reactions, and properties, integrating journal articles, patents, and scientific literature	Journal articles, patents, and literature	Nov 08 2024 287,000,000 ubstances,68,000,000 reactions and 48,000,000 bioactivities	Synthetic chemistry, medicinal chemistry, material science research, support for chemical discovery, and synthesis planning	https://www.reaxys.com/

In the study of biomolecular interaction prediction, selecting evaluation metrics also plays a crucial role in assessing the performance of predictive AI models. Since prediction tasks in this field often involve complex biological mechanisms and large‐scale data analysis, relying on a single evaluation metric may not provide a comprehensive assessment of a model's strengths and weaknesses. Therefore, a carefully chosen combination of multiple evaluation metrics is essential, as it not only allows researchers to objectively evaluate the predictive capability of a model but also provides a scientific basis for further optimization and improvement. This review takes PPI as an example to list several commonly used evaluation metrics for model assessment (**Table** **2**).

**Table 2:** Evaluation metric of AI‐based biomolecular interaction prediction.

Evaluation Metric	Definition	Evaluation Criteria	Mathematical Formula	Value Range
Accuracy (ACC)^[^ ^72^ ^]^	Accuracy measures the proportion of correctly predicted samples, representing the ratio of correctly classified instances to the total number of instances.	Higher accuracy generally suggests better performance. However, in imbalanced datasets, this metric can be misleading since a model can achieve a high ACC by simply predicting the majority class.	$ACC=\frac{TP+TN}{TP+TN+FP+FN}$ ^a)^	[0,1] ↑
Matthews Correlation Coefficient (MCC)^[^ ^73^ ^]^	MCC measures the correlation between predicted and actual classifications. It is a balanced metric that remains effective even when the dataset is imbalanced.	MCC is a reliable metric for PPI prediction, especially in imbalanced datasets.	$MCC=\frac{TP\times TN-FP\times FN}{\sqrt{\left(TP+FP)(TP+FN)(TN+FP)(TN+FN\right)}}$	[‐1,1] ↑
F1‐Score^[^ ^74^ ^]^	F1‐score is the harmonic mean of precision and recall, balancing the trade‐off between these two metrics.	F1‐score is useful for imbalanced datasets, as a higher F1‐score indicates a better balance between recall and precision.	$F1=\frac{2\times Precision\times Recall}{Precision+Recall}$ ^b)^	[0,1] ↑
Area Under the Curve (AUC)^[^ ^75^ ^]^	AUC quantifies the model's ability to distinguish between positive and negative samples by measuring the area under the Receiver Operating Characteristic (ROC) curve.	A higher AUC value indicates a stronger ability to distinguish between interacting and non‐interacting pairs in PPI prediction.	$AUC=\int_{0}^{1}TPR\left(FPR\right)d\left(FPR\right)$ ^c)^	[0,1] ↑
Mean Squared Error (MSE)^[^ ^76^ ^]^	MSE measures the average squared difference between predicted values and actual values.	MSE is mainly used in regression tasks to measure numerical prediction accuracy. However, it is sensitive to outliers.	$MSE=\frac{1}{n}\sum_{i=1}^{n}{\left(y_{i}-{\hat{y}}_{i}\right)}^{2}$ ^d)^	[0,+∞) ↓
Average Precision (AP)^[^ ^77^ ^]^	AP evaluates the ranking performance of a model by computing the average precision at different recall levels.	A higher AP score suggests better ranking capability in PPI prediction.	$AP=\sum_{I}\left(R_{i}-R_{i-1}\right)P_{i}$ ^e)^	[0,1] ↑
Area Under Precision‐Recall Curve (AUPRC)^[^ ^78^ ^]^	AUPRC assesses the trade‐off between precision and recall, making it more suitable than AUC for imbalanced datasets.	AUPRC is particularly useful in PPI prediction when dealing with imbalanced datasets, as it focuses on the model's performance for the positive class.	$AUPRC=\int_{0}^{1}P\left(R\right)dR$ ^f)^	[0,1] ↑
TM‐score^[^ ^79^ ^]^	A length‐normalized metric assessing the global topological similarity between two protein structures.	Measures overall similarity between predicted and native complex structures. Robust to length variation.	$TM-score=max(\frac{1}{L_{target}}\sum_{i=1}^{L_{aligned}}\frac{1}{1+{\left(\frac{d_{i}}{d_{o}\left(L\right)}\right)}^{2}})$ ^g)^ *, where* $d_{0}\left(L\right)=1.24{\left(L-15\right)}^{\left(1/3\right)}-1.8$	(0,1] ↑
Z‐score^[^ ^80^ ^]^	Measures the deviation of a score from the mean of a background distribution in standard deviations.	Indicates statistical significance of the predicted interaction compared to a random background.	$Z=\frac{S-\mu }{\sigma }$ ^h)^	Typically > 1.5 is significant
Root‐mean‐square deviation (RMSD)^[^ ^81^ ^]^	Root‐mean‐square deviation of atomic distances between predicted and native structures.	Measures atomic‐level accuracy, especially at the binding interface.	$RMSD=\sqrt{\frac{1}{N}\sum_{i=1}^{N}∥x_{i}^{pred}-x_{i}^{true}{∥}^{2}}$	[0, +∞) ↓
(Interface RMSD) iRMSD^[^ ^82^ ^]^	Interface RMSD; RMSD calculated only over the interfacial residues involved in binding.	Focuses on structural accuracy at the interaction interface, a key metric in docking assessments.	$iRMSD=\sqrt{\frac{1}{N_{interface}}\sum_{i=1}^{N_{interface}}∥x_{i}^{pred}-x_{i}^{native}{∥}^{2}}$	[0, +∞), < 2.5 Å preferred
Fnat^[^ ^83^ ^]^	Fraction of native residue‐residue contacts preserved in the predicted complex structure.	Measures the extent to which interfacial residue contacts are retained in the prediction.	$Fnat=\frac{Number\;of\;native\;contacts\;in\;the\;prediction}{Total\;number\;of\;native\;contacts}$	[0, 1] ↑
(Local Distance Difference Test) lDDT^[^ ^84^ ^]^	Local Distance Difference Test: measures how well local inter‐atomic distances are preserved.	Evaluates local structural accuracy, especially near interaction interfaces.	$lDDT=\frac{1}{N}\sum_{i=1}^{N}\frac{1}{n_{i}}\sum_{j\varepsilon neighbors(i)}\delta \left(d_{ij}^{pred},d_{ij}^{ture}\right)$	[0, 1] ↑
DockQ score^[^ ^85^ ^]^	A composite score combining RMSD, interface RMSD (iRMSD), and fraction of native contacts (Fnat).	Provides an overall evaluation of docking accuracy, considering both geometric and interface features.	$DockQ=\frac{1}{1+{\left(\frac{iRMSD}{1.5}\right)}^{2}+{\left(\frac{RMSD}{8.5}\right)}^{2}+{\left(1-Fnat\right)}^{2}}$	[0, 1] ↑

^a)^ TP: True Positive – correctly predicted positive samples; TN: True Negative – correctly predicted negative samples; FP: False Positive – negative samples incorrectly predicted as positive; FN: False Negative – positive samples incorrectly predicted as negative;

^b)^ Precision = TP/(TP+FP): how many predicted positives are actually positive; Recall = TP/(TP+FN): how many actual positives are correctly identified;

^c)^ TPR = TP/(TP+FN),True Positive Rate; FPR = FP/(FP+TN),False Positive Rate;

^d)^ n: number of samples; *y*_i_: ground truth value of the i‐th sample; ${\hat{y}}_{i}$: predicted value of the i‐th sample;

^e)^ *R*_i_: Recall at threshold n; *P*_i_: Precision at threshold n;

^f)^ P(R) denotes the precision as a function of recall;

^g)^ *L*_target_: the number of residues in the target (native) protein structure; used to normalize the TM‐score so it is independent of protein size. *L*_aligned_: the number of residue pairs that are successfully aligned between the predicted and target structures. *d*_i_: the Euclidean distance between the ii‐th pair of aligned residues;

^h)^ S: the observed score for the model or alignment of interest. μ: the mean score of the background. σ: the standard deviation of the background scores.

## 3 Popular AI Algorithms in Predicting Biomolecular Interactions

### 3.1 Protein‐Protein Interactions

Protein‐protein interactions (PPIs) are fundamental components of cellular activities, playing a vital role in various biological functions. These interactions drive proteins to create complex and dynamic networks that regulate biological processes, including signal transduction and enzyme catalysis reactions.^[^ ^87^, ^88^ ^]^ An in‐depth study of PPIs not only unravels the complex regulatory mechanisms of biological systems but also holds significant value for discovering novel therapeutic targets. With the rapid development of deep learning technologies, an increasing number of studies are focused on applying them to PPI prediction. The introduction of deep learning offers transformative possibilities for PPI prediction, promising to drive precise, efficient, and insightful computational methods, leading to new breakthroughs in this area.^[^ ^89^ ^]^

Traditional computational approaches to PPI prediction, such as molecular docking, statistical potentials, and co‐evolutionary analyses, have provided valuable insights but also encounter notable limitations.^[^ ^89^, ^90^, ^91^ ^]^ These methods often rely on handcrafted features, rigid structural assumptions, or sequence homology, which may not capture the full complexity and variability of protein interfaces. Additionally, many traditional models struggle to generalize across different protein families or accommodate noisy or incomplete data, limiting their predictive power in large‐scale or real‐world settings. The advent of CNNs has created new opportunities to address these challenges. By learning hierarchical representations directly from raw input data, such as protein sequences, distance maps, or 3D structures, CNNs can automatically extract local spatial features relevant to binding and interaction.^[^ ^92^ ^]^ This ability to learn discriminative patterns without manual feature engineering allows CNNs to model complex interface characteristics and enhances generalization across diverse protein types. As a result, CNN‐based models have demonstrated improved accuracy in identifying interacting residues, classifying binding sites, and predicting interaction likelihoods, making them a valuable tool in modern PPI research.

DPPI (Table 3) utilizes a deep CNN architecture, concentrating on extracting local features from protein profiles. It is the first work to implement deep learning techniques for PPI prediction and has achieved state‐of‐the‐art performance in binary prediction tasks.^[^ ^10^ ^]^ However, this method requires extensive data preprocessing and fails to capture the contextual and sequential features of protein sequences. In contrast, DNN‐PPI(Table 3) employs a structure with two separate CNN encoders; however, it does not incorporate physicochemical properties into the amino acid representation and lacks a Siamese learning architecture to fully capture the pairwise relationships between sequences.^[^ ^93^, ^94^ ^]^

**Figure 2:**
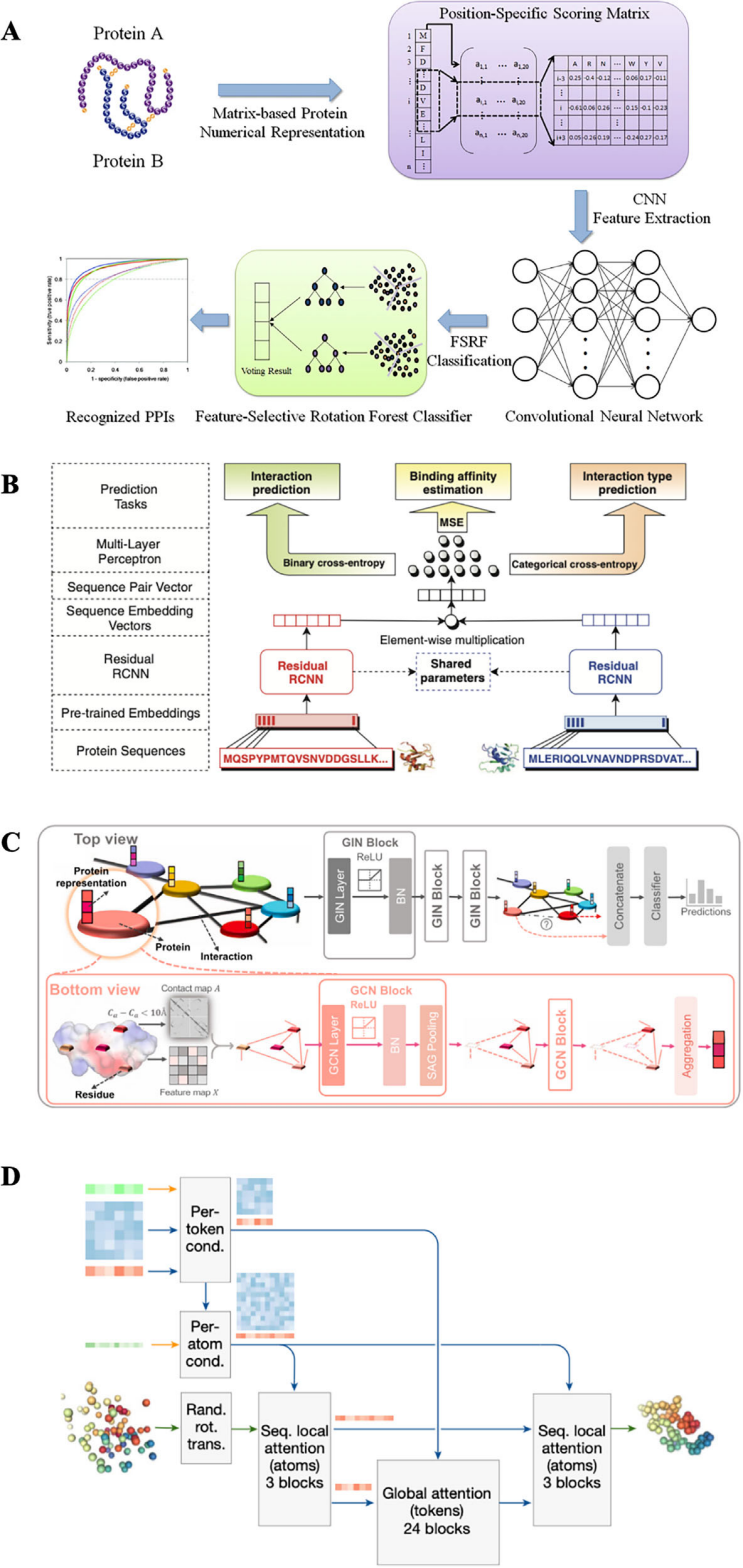
Deep learning‐based models for Protein‐Protein interaction prediction. A) Architecture of CNN‐FSRF. The framework begins with two protein sequences, which are numerically encoded using position‐specific scoring matrices. These matrix‐based representations are then processed through a CNN to extract interaction‐relevant features. The extracted features are subsequently input into a feature‐selective rotation forest classifier for final prediction. The ensemble classification mechanism outputs the voting result and identifies the PPIs, as illustrated by the ROC performance curves. Reproduced with permission from Wang et al.^[^ ^95^ ^]^ Copyright 2019 Spring Nature. B) Architecture of PIPR (Protein–Protein Interaction Prediction Based on Siamese Residual RCNN). The model takes pairs of protein sequences as input, which are first encoded using pre‐trained embeddings. These embeddings are then processed by a shared Residual RCNN module to extract deep sequence representations for each protein. The resulting embeddings are combined via element‐wise multiplication and fed into a multi‐layer perceptron for three different prediction tasks: binary interaction prediction, binding affinity estimation, and interaction type classification. The architecture enables shared representation learning while optimizing for multiple biologically relevant outputs. Reproduced with permission from Chen et al.^[^ ^93^ ^]^ Copyright 2019 Oxford University Press. C) Architecture of HIGH‐PPI(Hierarchical Graph‐based deep learning framework for Protein‐Protein Interaction prediction). The top view illustrates a PPI network where each node represents a protein with learned structural features, and edges indicate interactions. Protein representations are learned using multiple GIN blocks, and the resulting features are concatenated and passed to a classifier for final prediction. The bottom view shows the construction of protein representations at the residue level. Residues are connected based on spatial proximity to generate a residue‐level contact graph, where feature maps and contact maps are input into GCN blocks with SAG pooling and batch normalization. The extracted residue‐level features are aggregated to form protein‐level embeddings used in the top graph. Reproduced with permission from Gao et al.^[^ ^98^ ^]^ Copyright 2023 Spring Nature. D) Architecture of AlphaFold 3. This model integrates both atomic‐level and token‐level representations using a combination of local and global attention mechanisms. Atomic coordinates are subjected to random rotational transformations for invariance, followed by sequential local attention layers to extract neighborhood‐level features. These features are combined with per‐atom and per‐token conditional encodings and passed through a global attention module composed of 24 attention blocks. The final representation is refined through additional local attention blocks to predict or reconstruct 3D biomolecular structures with enhanced spatial and contextual accuracy. Reproduced with permission from Abramson et al.^[^ ^17^ ^]^ Copyright 2024 Spring Nature.

Wang *et al.* proposed a novel computational approach, CNN‐FSRF, which combines the advantages of deep learning and traditional machine learning to achieve efficient prediction of protein‐protein interactions (**Figure** **2**A, Table 3).^[^ ^96^ ^]^ The core innovation of CNN‐FSRF lies in its unique feature extraction and classification strategy. First, this method utilizes the Position‐Specific Scoring Matrix (PSSM) to convert the amino acid sequence of a protein into a numerical matrix that contains evolutionary information, providing biologically meaningful input data for subsequent deep learning modeling. Then, the model employs a CNN to automatically extract deep‐sequence features of proteins. Compared to traditional feature engineering methods, CNN can adaptively learn key patterns from the data without human intervention, reducing biases caused by manual feature selection while effectively filtering out redundant information, which makes the extracted features more biologically interpretable. To further optimize classification performance, the model applies an FSRF to denoise the features extracted by the CNN and performs the final prediction. By incorporating an improved rotation forest approach, FSRF not only enhances the robustness of the model but also reduces computational overhead, enabling high prediction accuracy while significantly improving prediction speed. Representations, or sliding window fragments, are inherently “locally aware.” Their convolutional kernels operate within a fixed receptive field, making it challenging to capture long‐range dependencies between residues that are distant in the sequence. In protein sequences, functionally related residues may be separated by considerable linear distances, and CNNs often struggle to effectively model such long‐range interactions. In contrast, Recurrent Neural Networks (RNNs), particularly architectures like Long Short‐Term Memory (LSTM) and Gated Recurrent Unit (GRU), are well‐suited for processing sequential data. They retain historical information and propagate it through subsequent steps, making them especially effective for modeling contextual dependencies within protein sequences. RNNs can learn semantic relationships between residues at different positions—for example, when an active site depends on a distant regulatory region—thereby capturing latent sequence‐level interactions.

PIPR (Protein–Protein Interaction Prediction Based on Siamese Residual RCNN) adopts a Siamese Residual RCNN (Residual Recurrent Convolutional Neural Network) architecture, relying solely on primary protein sequence information for PPI prediction(Figure 2B, Table 3).^[^ ^94^ ^]^ This model integrates the local feature extraction capabilities of CNNs with the contextual sequence processing power of RNNs, enabling it to effectively capture interaction‐related features within protein sequences. PIPR employs a Siamese network with shared parameters to model the interactions between protein pairs and utilizes a residual mechanism to mitigate the vanishing gradient problem in deep neural networks, thereby improving training stability and generalization performance. Additionally, its multi‐level feature aggregation mechanism allows the model to leverage both local and global sequence information, providing a more comprehensive representation for PPI prediction. Experimental evaluations demonstrate that PIPR outperforms various state‐of‐the‐art deep learning methods in binary PPI prediction and exhibits superior performance in the more challenging tasks of multi‐class interaction type prediction and binding affinity estimation. In the binary classification task, PIPR surpasses traditional methods such as SVM (Support Vector Machine), Random Forest, DNN‐PPI, and DPPI across multiple benchmark datasets, achieving higher prediction accuracy and F1‐score. Furthermore, Li *et al.* developed DELPHI (Table 3), which integrates a CNN and an RNN in an ensemble structure, further optimizing model performance through fine‐tuning.^[^ ^97^ ^]^ Compared to traditional methods, this model introduces three novel features: HSP (High‐coring segment pairs), position information, and ProtVec1D (a dimension‐reduced feature based on 3‐mer amino acid embeddings), achieving outstanding predictive performance across multiple datasets. DELPHI adopts a sequence feature‐driven approach for predicting PPI binding sites, with its primary advantage being that it does not rely on protein 3D structures; instead, it efficiently predicts based on sequence information, greatly expanding its applicability.

While CNNs and RNNs have achieved promising results in PPI prediction by extracting local sequence features and modeling contextual dependencies, they are inherently limited in capturing the topological structure of protein interaction networks and spatial relationships within molecular graphs. CNNs are primarily designed for grid‐like data and often struggle to model complex global dependencies, while RNNs are confined to linear sequence processing. However, many biological systems—such as protein‐protein interaction networks and molecular structures—are more naturally represented as graphs rather than sequences or grids. Graph Neural Networks, specifically designed to operate on graph‐structured data, offer a powerful alternative.^[^ ^97^ ^]^ By learning directly from nodes (e.g., proteins), edges (e.g., interactions), and overall network topology, GNNs can effectively model both local and global interaction patterns, providing complementary insights beyond those offered by CNNs and RNNs. In the context of PPI prediction, GNNs can capture not only whether two proteins interact but also how they engage within the broader network or structural context.

The HIGH‐PPI model consists of two GNN components, each designed to learn information at different levels (Figure 2C, Table 3).^[^ ^98^ ^]^ In this framework, proteins are represented as nodes and their interactions as edges in the PPI network. Through an end‐to‐end training mechanism, HIGH‐PPI establishes interactions across both levels, allowing individual protein representations to optimize PPI network predictions while also enabling PPI network learning to enhance protein structural representations. Jha *et al.* proposed a PPI prediction model based on GNNs(Table 3). This approach utilizes Graph Convolutional Networks (GCN) and Graph Attention Networks (GAT), integrating protein structural information with sequence features for PPI prediction.^[^ ^99^ ^]^ The model utilizes protein language models to extract features from amino acid sequences, enabling each node to obtain sequence‐based embeddings. This advancement enhances the model's comprehension of protein structure and functional relationships. This method simultaneously incorporates both sequence and structural information of proteins, addressing the limitations of traditional methods that depend solely on sequence features and improving the accuracy of PPI prediction.

While GNNs have demonstrated strong capabilities in modeling the structural and topological information of proteins, they still encounter challenges in capturing long‐range dependencies and global contextual relationships within sequences or 3D structures. Most GNNs depend on message passing between neighboring nodes, which can result in over‐smoothing and limited receptive fields, particularly in deep‐layer architectures.^[^ ^99^ ^]^ This limits their ability to model interactions between distant residues or across different domains of a protein. The Transformer model, based on a self‐attention mechanism, overcomes these limitations by directly modeling global dependencies, regardless of spatial or sequential distance. It can simultaneously learn relationships between all residues, capturing both local and long‐range interactions without the constraints of graph topology or sequential order. Moreover, the parallelizable architecture of Transformers significantly enhances computational efficiency, making them well‐suited for large‐scale protein datasets. In protein‐related tasks, Transformer encoders are typically employed to extract deep contextual representations from amino acid sequences or structure‐informed inputs, which have shown superior performance in protein structure prediction, binding site identification, and interaction modeling.

Significant advancements in biomedical research have occurred due to the development of sophisticated AI models, with one of the most groundbreaking innovations being AlphaFold 3 (Figure 2D, Table 3).^[^ ^17^ ^]^ The latest iteration of the renowned AlphaFold series, developed by DeepMind, represents a monumental leap in predicting biomolecular interactions with unprecedented accuracy. The overall architecture of AlphaFold 3 retains the fundamental principles established by AlphaFold 2, which involve both generating pairwise representations of chemical complexes through a network trunk and utilizing these representations to predict atomic‐level structures. The core innovation of AlphaFold 3 lies in adopting a diffusion generative module that directly predicts atomic coordinates, thereby eliminating the reliance on AlphaFold 2's complex frame‐based structural modules. One of the key improvements in AlphaFold 3 is the introduction of the Pairformer module, which replaces the Evoformer module used in AlphaFold 2 as the primary processing unit. The Pairformer operates exclusively on pairwise and single representations of chemical complexes, simplifying the complex multiple sequence alignment (MSA) processing utilized by AlphaFold 2. This module employs a lightweight MSA embedding mechanism, significantly reducing computational overhead by limiting the number of MSA blocks to just four. Another critical architectural feature of AlphaFold 3 is the introduction of the Diffusion Generative Module, which predicts 3D atomic coordinates using a multi‐scale diffusion approach. By gradually removing noise from the input coordinates, this module generates a distribution of potential molecular structures rather than a single deterministic result, enhancing both the accuracy and comprehensiveness of the predictions. The module focuses on refining local molecular structures at lower noise levels and addressing the global structure at higher noise levels.

Using molecules as inputs, a model trained on a vast dataset of known biomolecular interactions, can predict binding and long‐range interactions, which is particularly important for modeling complex molecular assemblies. In terms of precision, AlphaFold 3 outperforms specialized tools in multiple biomolecular interaction predictions. For instance, in protein‐ligand interaction predictions, AlphaFold 3 has achieved significantly higher accuracy compared to traditional docking tools, especially when the ligand RMSD (root mean square deviation) to the aligned binding pocket is less than 2 Å.

AlphaFold 3 demonstrates outstanding performance in predicting PPI, especially in accurately modeling complex protein assemblies. By utilizing a novel diffusion‐based model architecture, AlphaFold 3 effectively captures the 3D conformations of protein‐protein interactions, achieving significantly higher accuracy, particularly when predicting low‐homology protein complexes. When compared to AlphaFold‐Multimer 2.3, AlphaFold 3 reveals substantial advantages in PPI prediction, with notably superior accuracy in antibody‐antigen interaction modeling. AlphaFold 3's scalability allows it to manage a wide range of biomolecules found in the PDB. Importantly, it has excelled in predicting complex molecular interactions, such as protein–antibody interactions and protein‐RNA interactions, thereby opening new possibilities in drug discovery and disease research. In antiviral therapeutics, AlphaFold 3 has played a crucial role in predicting interactions between viral proteins and human host receptors. For instance, it accurately predicted the binding mode of the SARS‐CoV‐2 spike protein to the ACE2 receptor, contributing to the development of vaccines and antiviral drugs.^[^ ^100^, ^101^, ^102^, ^103^ ^]^ To overcome challenges associated with systems that lack sufficient co‐evolutionary information, a method involving cross‐linking mass spectrometry (XL‐MS) has been introduced. This approach, which models chemical crosslinkers as covalent ligands directly integrated into AlphaFold 3's prediction pipeline, allows for accurate predictions of complex biomolecular interactions, such as protein‐antibody complexes.^[^ ^104^ ^]^

One of the primary concerns with AlphaFold 3 is its handling of stereochemistry and molecular constraints. While the diffusion‐based approach enhances flexibility, it also introduces chirality errors and atomic clashes, particularly in complex biomolecular systems. The model struggles to maintain the correct stereochemical configuration in some predictions, as evidenced by a 4.4% chirality violation rate in benchmark tests. Additionally, AlphaFold 3 occasionally produces overlapping atomic structures, especially in protein‐nucleic acid complexes with large nucleotides (>100) and extensive residues (>2, 000), suggesting limitations in its ability to enforce steric constraints. These issues indicate that while AlphaFold 3 can generate highly plausible structures, it sometimes fails to maintain chemically and physically valid conformations. Another major limitation is hallucination, a common issue in generative models where structurally undefined or disordered regions are falsely predicted as ordered structures. AlphaFold 3's reliance on diffusion means it may generate overly confident but incorrect conformations in flexible regions, particularly for intrinsically disordered proteins (IDPs) or flexible loop regions. While the model includes a cross‐distillation process to mitigate hallucinations—training on AlphaFold‐Multimer v2. 3 outputs where disordered regions are explicitly represented—some errors persist. This could lead to misinterpretations in functional studies that rely on AlphaFold 3 predictions for structural insights. The model also struggles with conformational dynamics. Like its predecessors, AlphaFold 3 is designed to predict static structures, often corresponding to crystallographic or cryo‐EM‐derived conformations. However, many biomolecular interactions involve dynamic conformational changes that cannot be fully captured by a single static model. This limitation is particularly evident in multi‐state proteins, such as ubiquitin ligases, where AlphaFold 3 consistently predicts a closed conformation even when an open state is experimentally observed. Unlike physics‐based molecular dynamics simulations, which can explore multiple conformations over time, AlphaFold 3 is limited to discrete, single‐structure predictions, reducing its utility in studying allosteric regulation and ligand‐induced conformational shifts.

### 3.2 Protein–Nucleic Acid Interactions

Protein‐nucleic acid interactions are essential in biological processes such as genetic regulation, transcription, translation, and RNA processing. However, due to the high variability and flexibility of nucleic acids, predicting the structure of protein‐nucleic acid complexes remains more challenging than predicting protein‐protein interactions. Traditional methods rely on molecular docking strategies, in which the 3D structures of proteins and nucleic acids are predicted separately before computational docking is used to determine potential binding modes. These approaches often require known homologous structural templates or optimized docking scoring functions, making it difficult to efficiently and accurately predict novel protein‐nucleic acid interaction patterns. The emergence of deep learning has provided a new solution for predicting protein‐nucleic acid interactions. In recent years, deep learning models such as AlphaFold and RoseTTAFold have achieved groundbreaking success in protein structure prediction, accurately predicting 3D protein structures based on MSA information through end‐to‐end learning. This success has inspired researchers to extend similar approaches to predicting protein‐nucleic acid complexes. Compared to traditional methods, deep learning can learn complex interaction patterns from extensive structural data and directly integrate protein‐nucleic acid interactions during the prediction process, rather than modeling them separately and docking afterward.

**Figure 3:**
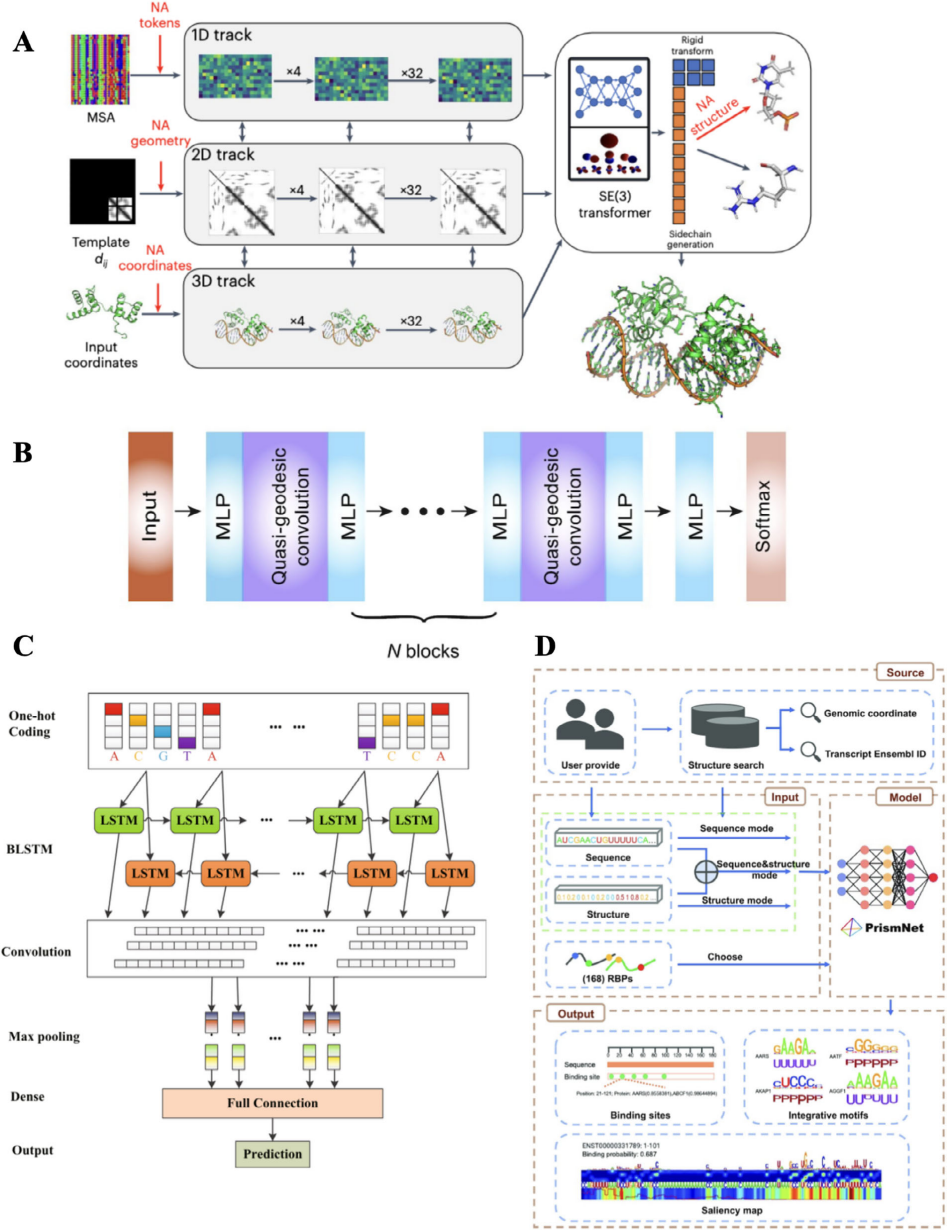
Deep learning‐based models for Protein‐Nucleic Acid interaction prediction. A) Architecture of RoseTTAFoldNA. The model employs a three‐track architecture to jointly learn sequence, geometric, and coordinate information from both nucleic acids and proteins. The 1D track processes nucleic acid multiple sequence alignments to extract sequence‐level representations. The 2D track models the nucleic acid geometry using pairwise templates, while the 3D track processes atomic coordinates from the input structure. These tracks exchange information across layers, enabling integrated learning. A SE(3)‐equivariant transformer is used to generate final atomic‐level structures and sidechains, producing high‐resolution 3D models of nucleic acid–protein complexes. Reproduced with permission from Baek et al.^[^ ^105^ ^]^ Copyright 2023 Spring Nature. B) Architecture of GeoBind(Geometric Binding Site Prediction). The model takes input features from biomolecular surfaces and processes them through multiple stacked blocks, each consisting of an MLP followed by quasi‐geodesic convolution operations. These convolutions account for surface geometry by approximating geodesic distances on molecular surfaces. After passing through N such blocks, the extracted features are further transformed by additional MLP layers, followed by a softmax output layer to produce final predictions, such as binding site classification or surface segmentation. Reproduced with permission from Li et al.^[^ ^106^ ^]^ Copyright 2023 Oxford University Press on behalf of Nucleic Acids Research. C) Architecture of DeepSite. The model begins with one‐hot encoding of nucleotide sequences (e.g., A, T, C, G), which are fed into a bidirectional LSTM layer to capture contextual dependencies in both forward and backward directions. The output of the BLSTM is passed through convolutional layers to extract spatial patterns and local motifs. Following convolution, max pooling is applied to reduce dimensionality and emphasize dominant features. These features are then passed through dense (fully connected) layers, leading to the final prediction output. Reproduced with permission from Zhang et al.^[^ ^107^ ^]^ Copyright 2019, Springer‐Verlag GmbH Germany, part of Springer Nature. D) Architecture of PrismNet. The input can be provided by users through genomic coordinates or transcript Ensembl IDs, which are used to retrieve both RNA sequence and predicted structural information. PrismNet supports three input modes: sequence‐only, structure‐only, and combined sequence‐structure mode. Users can select any of 168 RBPs for analysis. The model uses a neural network to integrate the selected features and predict RBP binding sites. The outputs include predicted binding sites along the RNA sequence, integrative sequence‐structure motifs, and saliency maps that highlight the most informative positions contributing to the prediction. Reproduced with permission from Xu et al.^[^ ^137^ ^]^ Copyright 2023, Oxford University Press on behalf of Nucleic Acids Research.

Using RoseTTAFold, the research team developed RoseTTAFoldNA Modifications tailored for nucleic acids include nucleic acid‐specific geometric representations, expanded interaction features, and enhanced fine‐grained modeling of nucleic acid structures (**Figure** **3**A, Table 3).^[^ ^105^ ^]^ Experimental results demonstrate that RoseTTAFoldNA significantly outperforms existing methods in predicting protein‐nucleic acid complexes, particularly in cases lacking known homologous structures. In a test set of 224 monomeric protein‐nucleic acid complexes, the model attained an average lDDT score of 0.73, with 29% of predictions showing lDDT > 0.8 and 45% of models exhibiting FNAT > 0.5. Even in 33 cases without homologous complexes, the method maintained an accuracy of lDDT = 0.68 and successfully predicted binding interfaces. For 161 multi‐subunit protein‐nucleic acid complexes, RoseTTAFoldNA achieved accuracy comparable to monomeric complexes, successfully capturing protein‐induced DNA bending. It surpassed approaches that rely on separately modeling individual components, followed by docking.

Despite its breakthroughs in predicting protein‐nucleic acid complexes, RoseTTAFoldNA still faces certain limitations. First, compared to AlphaFold's high precision in protein structure predictions, RoseTTAFoldNA's overall accuracy remains lower. While the model's average lDDT score of 0.73 demonstrates strong performance in predicting protein‐nucleic acid interactions, it does not yet match the precision achieved in protein monomer structure prediction. Additionally, the dataset of protein‐nucleic acid complexes is significantly smaller than that of protein structures, resulting in a relatively limited training dataset that affects the model's generalizability. This data scarcity particularly impacts its ability to predict novel protein‐nucleic acid complexes without homologous structures, diminishing prediction accuracy when dealing with rare or highly dynamic protein‐nucleic acid complexes. Stability remains a challenge when predicting large, multi‐domain proteins or extensive RNA/DNA complexes. For long‐chain RNAs, large single‐stranded DNAs, or supercoiled DNAs, the model's accuracy decreases, often leading to binding interface shifts or incorrect protein conformations. Furthermore, while RoseTTAFoldNA can predict protein‐induced DNA bending to some extent, it struggles with more complex DNA conformational changes, such as supercoiling, which may lead to inaccurate predictions for certain transcription factor‐DNA complexes. In RNA structure prediction, although RoseTTAFoldNA outperforms traditional methods, it still lags behind specialized deep learning models such as DeepFoldRNA and AIchemy_RNA in CASP15 assessments, indicating room for improvement in modeling RNA tertiary structures.

GeoBind uses geometric deep learning to predict nucleic acid binding sites on protein surfaces through a segmentation approach (Figure 3B, Table 3).^[^ ^106^ ^]^ This method processes protein point cloud data, constructs a local reference frame (LRF), and employs a quasi‐geodesic convolutional network to learn high‐level representations of the protein surface. Compared to existing methods, GeoBind offers a more complete analysis of protein‐nucleic acid interaction patterns and shows superior performance across multiple benchmark datasets. Furthermore, GeoBind is not restricted to monomeric proteins; it can also predict binding interfaces in multimeric protein complexes, thereby expanding its applicability in biological systems. Zhang *et al.* proposed DeepSite, a deep learning model that combines a bidirectional long short‐term memory network (BLSTM) with a CNN for predicting DNA‐protein binding sites (Figure 3C, Table 3).^[^ ^107^ ^]^ The model leverages BLSTM to capture long‐range dependencies in DNA sequences while integrating CNN to learn local sequence features, thereby enhancing the recognition of DNA binding sites. DeepSite consists of six layers: an input layer, a BLSTM layer, a CNN layer, a pooling layer, a fully connected layer, and an output layer. The BLSTM layer captures specific patterns that span long distances in DNA sequences, while the CNN is responsible for extracting local features, enabling the model to understand long‐range dependencies and effectively identify local sequence information, ultimately improving prediction accuracy. Additionally, Xu *et al.* proposed PrismNet, a computational model based on DNN specifically designed for predicting dynamic protein‐RNA (RBP‐RNA) interactions (Figure 3D, Table 3).^[^ ^108^ ^]^ Unlike traditional prediction methods, PrismNet integrates experimentally determined RNA secondary structure data with RBP binding data, enabling more accurate predictions of RBP binding under various cellular conditions. The key innovation of this model lies in its integration of RNA sequence information with in vivo RNA structural data, along with the use of an attention mechanism, allowing it to precisely identify the key nucleotides involved in RBP binding. PrismNet's predictions indicate that RNA structure plays a crucial role in determining dynamic RBP binding sites. Additionally, the study identified structural variation‐associated sites known as riboSNitches, which may influence RBP binding and are linked to human genetic diseases.

### 3.3 Protein–Small Molecule Interactions

With the rapid advancement of biotechnology, accurately designing interactions between proteins and small molecules has become a critical challenge in molecular biology and drug development. In particular, the field of designing small molecules to activate protein functions has long struggled with the difficulty of accurately regulating protein‐protein interactions within complex cellular environments. Therefore, developing protein‐small molecule complexes with high affinity and selectivity not only enhances the precision of cell therapies but also drives advancements in novel biosensors and synthetic biology. Although existing computational design methods, such as those based on natural amino acid combinations and other structural prediction approaches, have made some progress in protein design, they still encounter numerous challenges in designing protein‐small molecule complexes. The complexity of protein‐small molecule binding interfaces and the lack of sufficient structural data hinder traditional methods from accurately capturing the binding characteristics between small molecules and proteins.

**Figure 4:**
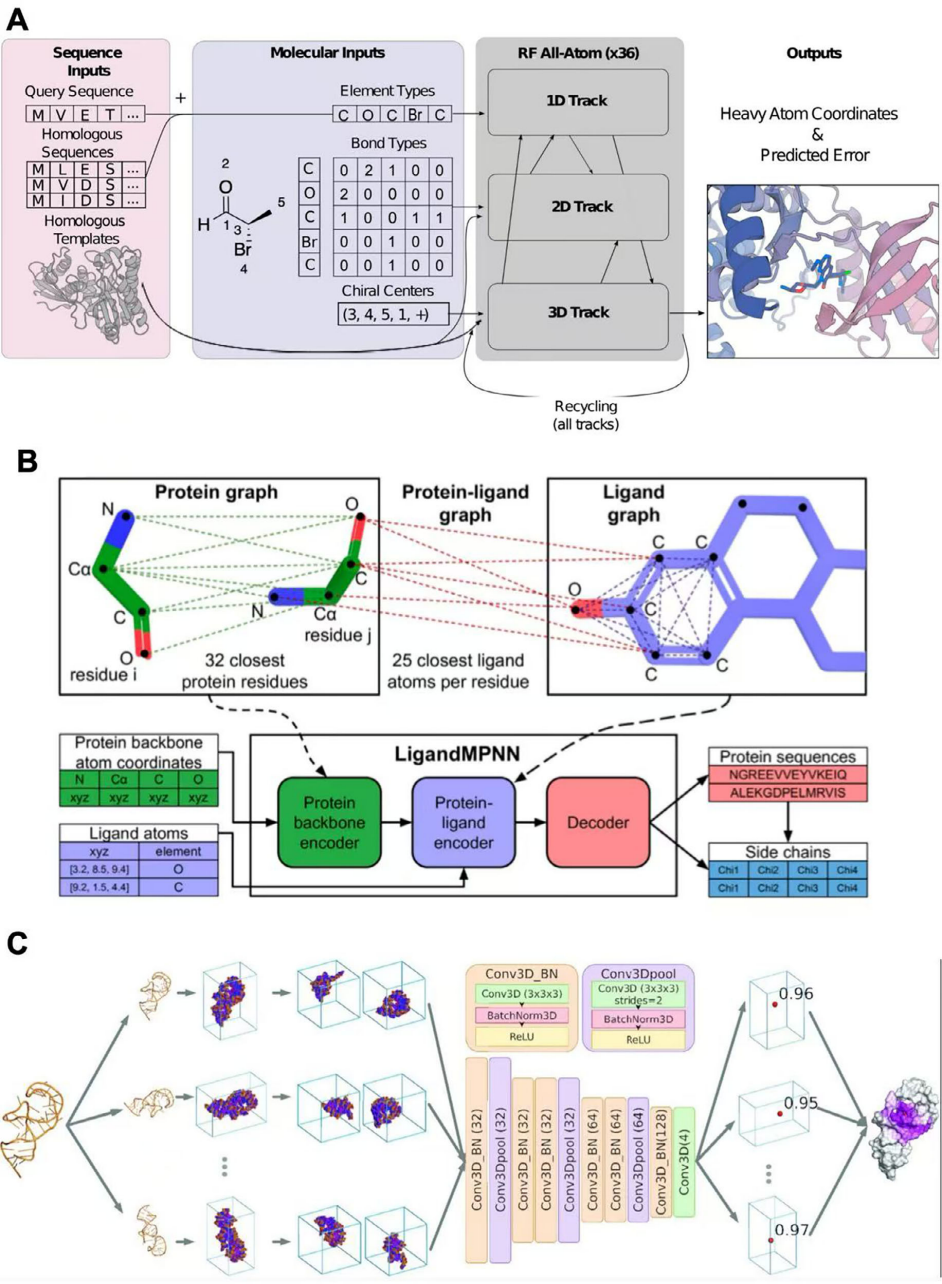
Deep learning‐based models for predicting Protein‐Small molecule interactions and Nucleic Acid‐Small molecule interactions. A) Architecture of RoseTTAFold All‐Atom. The model architecture includes three input modules: sequence inputs, molecular inputs, and structural templates. These inputs are processed by a 36‐layer main network with 1D, 2D, and 3D tracks, which exchange and recycle information across tracks. The final output includes heavy atom coordinates and confidence estimates for the modeled structure. Reproduced with permission from Krishna et al.^[^ ^109^ ^]^ Copyright 2024, The American Association for the Advancement of Science. B) Architecture of LigandMPNN. The model represents the protein–ligand system as three interconnected graphs: a protein graph (left), a ligand graph (right), and a protein–ligand interaction graph (center). The protein graph includes backbone atoms and connects each residue to its 32 closest residues. The ligand graph models atomic connectivity within the small molecule. The protein–ligand graph links each protein residue to its 25 closest ligand atoms. These spatial and structural relationships are encoded through a message‐passing framework. The architecture includes three modules: a protein backbone encoder that processes atom coordinates, a protein–ligand encoder that captures cross‐graph interactions, and a decoder that integrates information from protein sequences and predicts side‐chain conformations for the binding interface. Reproduced with permission from Dauparas et al.^[^ ^111^ ^]^ Copyright 2025 Springer Nature. C) Architecture of BiteNet_N_. The model processes RNA 3D structures by generating multiple local cubic regions centered around different positions of the molecule. Each voxel contains atomic density information encoded into a 3D grid. These voxelized structures are passed through a 3D‐CNN, which consists of stacked Conv3D layers, batch normalization, ReLU activations, and down‐sampling operations. The final layer predicts binding site confidence scores for each region. High‐scoring voxels are aggregated and mapped back to the RNA surface to identify potential small‐molecule binding pockets. Reproduced with permission from Kozlovskii et al.^[^ ^112^ ^]^ Copyright 2021, Oxford University Press on behalf of NAR Genomics and Bioinformatics.

Krishna *et al.* proposed RoseTTAFold All‐Atom, a deep neural network‐based biomolecular modeling method designed for high‐precision modeling of complete biological assemblies, including proteins, nucleic acids, small molecules, metal ions, and covalent bond modifications(**Figure** **4**A, Table 3).^[^ ^109^ ^]^ This model extends RoseTTAFold and AlphaFold2, overcoming the limitations of existing methods in dealing with small molecules, metal ions, and covalent modifications, thereby enabling the prediction of more complex biomolecular interactions. RoseTTAFold All‐Atom integrates sequence and structural information, optimizing 3D modeling through a deep learning network based on input protein and nucleic acid sequences, as well as small molecule structures, which improves prediction accuracy and applicability. Additionally, the study further developed RFdiffusion All‐Atom, fine‐tuning RoseTTAFold All‐Atom through a diffusion denoising task, allowing it to directly generate protein structures around small molecules. This approach successfully designed and validated proteins capable of binding to the cardiotonic drug digoxigenin, the enzymatic cofactor heme, and optically active bilin molecules.

Traditional protein sequence design methods, such as Rosetta and ProteinMPNN, primarily optimize sequences based on the protein backbone structure but do not accurately account for non‐protein atomic interactions and environments.^[^ ^110^ ^]^ This limitation restricts their applications in enzyme design, protein‐small molecule binding, nucleic acid‐binding protein design, and metal‐binding protein design. To address this issue, Dauparas *et al.* developed LigandMPNN(Ligand Message Passing Neural Network), a deep learning‐based protein sequence design method that can explicitly model non‐protein atomic environments, including small molecules, nucleotides, and metals, to optimize functional protein design(Figure 4B, Table 3).^[^ ^111^ ^]^ LigandMPNN introduces a protein‐ligand graph that captures not only the local conformational information of proteins but also integrates the geometric and chemical features of small molecules, thereby improving the accuracy of sequence prediction. The model's core consists of three modules: the protein backbone encoder, the protein‐ligand encoder, and the decoder. The encoders process the protein backbone and ligand geometry to capture the complex interactions between proteins and ligands, while the decoder autoregressively predicts the protein sequence and side‐chain torsion angles, generating a complete protein structure. To enhance performance, the model incorporates ligand geometry encoding and contextual augmentation strategies, further enriching interaction information.

The research team evaluated LigandMPNN's performance across multiple datasets and compared it with Rosetta and ProteinMPNN. Results showed that LigandMPNN significantly outperformed other methods in sequence recovery rates for residues involved in small molecule binding, nucleotide binding, and metal binding. To further assess LigandMPNN's reliability, the research team conducted side‐chain conformation prediction experiments. The results showed that LigandMPNN outperformed Rosetta and ProteinMPNN in chi1 and chi2 dihedral angle recovery rates, with predicted side‐chain conformations closely matching crystal structures. Additionally, LigandMPNN demonstrated success in various experimental protein‐small molecule binding design tasks. For example, researchers redesigned proteins binding to the muscle relaxant Rocuronium and bile acid Cholic acid using LigandMPNN, significantly improving Rocuronium‐binding protein affinity and increasing Cholic acid‐binding protein affinity by 100‐fold, further validating LigandMPNN's practical value in optimizing protein‐small molecule interactions. Despite its remarkable advancements, LigandMPNN still has certain limitations. First, it relies on existing protein‐ligand complex data for training, and since structural data for protein‐small molecule complexes are relatively scarce, the model's performance may be affected when handling rare ligands or novel compounds. Second, while LigandMPNN can predict side‐chain conformations, its performance in predicting higher‐order dihedral angles (such as chi3 and chi4) still requires improvement. Moreover, studies indicate that LigandMPNN depends on chemical element type input when predicting metal‐binding sites, and its performance significantly declines when this information is removed. This suggests that the model still requires deeper physicochemical constraints to enhance its understanding of non‐protein environments.

### 3.4 Nucleic Acid–Small Molecule Interactions

In recent years, nucleic acid molecules, particularly RNA, have received growing attention as drug targets, with RNA‐small molecule interactions showing significant potential in antiviral therapies, antibiotics, and gene regulation. However, due to the dynamic conformational nature of RNA molecules and the complexity of their binding sites, existing computational prediction methods continue to face many limitations. Specifically, there are relatively few methods for detecting RNA binding sites, and most rely on heuristic rules or small‐scale machine learning models. Consequently, developing efficient and accurate RNA‐small molecule binding site prediction tools is essential for RNA‐targeted drug discovery.

BiteNet_N_ is a deep learning‐based method for predicting binding sites that models nucleic acid‐small molecule interactions in three dimensions, CNN(Figure 4C, Table 3).^[^ ^112^ ^]^ The research team constructed a dataset containing ≈2000 nucleic acid‐small molecule complexes and employed a voxel‐based approach to represent nucleic acid structures. Each voxel represents a 1Å^3^ cube and stores multi‐channel atomic density information to capture the geometric features of nucleic acid binding sites. Experimental results demonstrate that BiteNet_N_ can accurately predict small‐molecule binding sites across various nucleic acid structures, including RNA, DNA, and their different conformations, such as A‐form double helices, B‐form double helices, Z‐form double helices, triple helices, and quadruple helices. To further validate BiteNet_N_'s applicability in real biological systems, the research team conducted two case studies on HIV‐1 TAR RNA and an ATP aptamer. In the HIV‐1 TAR RNA study, BiteNet_N_ successfully identified binding sites across different conformations, demonstrating its adaptability to RNA structural flexibility. Additionally, in molecular dynamics simulations of the ATP aptamer, the method effectively distinguished changes in binding site states between wild‐type and mutant variants, accurately reflecting experimentally observed binding trends. These findings highlight BiteNet_N_'s potential for studying nucleic acid conformational changes and its broader applicability in RNA‐targeted drug discovery.

Wang *et al.* proposed MultiModRLBP (Multi‐Modal RNA‐Small Molecule Ligand Binding Sites Prediction), a deep learning‐based multi‐modal RNA‐small molecule ligand binding site prediction model.^[^ ^113^ ^]^ MultiModRLBP employs a multi‐modal feature fusion strategy, integrating nucleotide‐level 3D structural properties of RNA molecules, relationship graphs based on overall RNA structures, and rich RNA semantic information to enhance prediction accuracy. The research team collected 851 RNA‐small molecule interaction data sets from the RNAglib dataset and the RLBind training set. Unlike traditional training sets, this dataset expands the research scope by including RNA complexes with the same RNA sequence but different binding sites due to structural variations or different ligand interactions. This enables the model to more accurately capture subtle RNA structural changes and improve its ability to distinguish differences between similar RNA conformations. Further experiments demonstrated that MultiModRLBP is particularly effective in predicting distant binding sites, which may be attributed to its capability to process complex RNA structural relationships and the RNABert (RNA Bidirectional Encoder Representations from Transformers) module's ability to extract global semantic information from RNA.^[^ ^114^ ^]^ Additionally, the model exhibited differences in performance when predicting metal ion and non‐metal ion binding sites, with better accuracy for non‐metallic ligands. Researchers speculate that this may be due to non‐metal ion‐binding RNA typically forming more well‐defined binding pockets, whereas metal ion binding sites tend to be more exposed.

In recent years, the application of deep learning in predicting biomolecular interactions has achieved significant advancements, offering powerful tools to uncover complex molecular relationships. However, the impact of these predictive approaches extends beyond understanding biological mechanisms, opening new avenues for synthetic biology and cellular engineering. For instance, Baker *et al.* developed a modular synthetic receptor system (SNIPR) that demonstrates how artificially designed signaling mechanisms can be utilized to detect soluble ligands via endocytosis and activate specific cellular functions.^[^ ^115^ ^]^ Snakebite envenoming remains a devastating tropical disease. Baker *et al.* developed a de novo designed synthetic protein to neutralize snake venom toxins, based on a SNIPR.^[^ ^116^ ^]^ This system is capable of detecting soluble ligands via endocytosis and activating specific cellular functions. The non‐conventional endocytosis‐dependent signaling mechanism of this receptor system overcomes the limitations of existing signaling pathways and provides a novel tool for constructing modular signaling networks. This innovative approach can be applied to targeted cellular responses in cancer therapy, offering new potential for cancer treatment and synthetic biology. Inflammation is closely associated with autoimmune responses and cancer, with its core mechanism involving a protein called the tumor necrosis factor receptor (TNFR), which serves as a target for many therapeutic drugs. Current drugs used to treat inflammation, such as Enbrel, have shown some efficacy. However, Matthias *et al.* demonstrated that AI‐synthesized proteins exhibit higher affinity and specificity for TNFR, highlighting their potential advantages in targeted therapy.^[^ ^117^ ^]^ Neurodegenerative diseases, particularly Alzheimer's disease, have garnered significant attention due to their association with the formation of amyloid fibrils. The process of amyloid fibril formation involves various proteins, notably Amyloid β and Tau, which interact with each other to form these fibrils. Sahtoe *et al.* designed a novel protein that binds to the disordered regions of these proteins, effectively inhibiting the formation of amyloid proteins and thereby preventing the onset of Alzheimer's disease.^[^ ^118^ ^]^

**Table 3:** Representative model of AI‐based biomolecular interaction prediction.

Model	Backbone	Data	Application	Result	Advantages
DPPI^[^ ^10^ ^]^	Siamese‐like CNN + Random Projection	Human and yeast PPI datasets (HIPPIE v1.2, DIP core set), HINT database (multiple species)	Sequence‐based PPI prediction, applicable to protein‐protein interaction recognition	ACC = 0.946 F1 = 0.922, AUPRC = 0.967	Uses sequence information for PPI prediction, reducing experimental noise; Introduces random projection module for better homodimer detection; More computationally efficient than traditional SVM‐based methods
DNN‐PPI^[^ ^94^ ^]^	CNN + LSTM	Pan's human PPI dataset, HPRD, DIP, HIPPIE, inWeb_inbiomap, E. coli, Drosophila, C. elegans, Mus Musculus datasets	Deep neural network‐based PPI prediction, applicable to cross‐species PPI prediction	ACC = 0.988, F1 = 0.988, MCC = 0.979 (Benchmark dataset); ACC = 0.979 (HPRD), ACC = 0.943 (DIP), ACC = 0.961 (HIPPIE HQ), ACC = 0.934 (HIPPIE LQ), ACC = 0.931 (inWeb_inbiomap HQ), ACC = 0.928 (inWeb_inbiomap LQ)	No need for handcrafted feature extraction, reducing preprocessing effort; Automatically learns both local and global sequence information; Strong generalization ability, applicable to cross‐species PPI prediction
AlphaFold^[^ ^14^ ^]^	Deep Residual CNN + Potential Optimization	PDB database, CASP13 assessment data	Predicts the 3D structure of single proteins, improving accuracy in structural biology modeling	TM‐score = 0.700 (FM dataset), Z‐score = 52.8 (FM category), Z‐score = 68.3 (FM + TBM/FM category)	Distance‐based potential method provides richer structural information than contact‐based methods; Effective for free modelling (FM) tasks, significantly outperforming other methods in CASP13; Achieves high‐accuracy structure prediction without template information.
AlphaFold 2^[^ ^14^ ^]^	Evoformer + Structure Module (Transformer‐based deep learning model)	Trained using MSA and PDB database, incorporating physical and biological constraints	Predicts protein 3D structures without homologous templates, enabling high‐accuracy structural modeling	CASP14 evaluation: Backbone RMSD = 0.96Å, All‐atom RMSD = 1.5Å, lDDT = 92.4, TM‐score = 0.93	First model to achieve near‐atomic accuracy even for proteins with no homologous structures, significantly surpassing existing methods
AlphaFold 3^[^ ^17^ ^]^	Pairformer + Diffusion Module	PDB database, CASP15 dataset, PoseBusters dataset	Predicts high‐accuracy 3D structures for protein‐protein, protein‐nucleic acid, and protein‐ligand complexes	CASP15 Evaluation: LDDT = 0.924, RMSD = 1.5Å; PoseBusters Evaluation: Pocket‐aligned ligand RMSD < 2Å; Protein‐Protein DockQ Score: Improved by 18%	Supports protein‐protein, protein‐nucleic acid, and protein‐ligand complex prediction; Eliminates MSA‐heavy processing for improved efficiency; Diffusion model improves generalization by reducing reliance on chemical constraints
CNN‐FSRF^[^ ^95^ ^]^	CNN + Feature‐Selective Rotation Forest (FSRF)	Yeast dataset (DIP) and Helicobacter pylori dataset	Sequence‐based PPI prediction, applicable to binary classification, improves accuracy and generalization ability	ACC = 0.978, F1 = 0.978, MCC = 0.956, AUC = 0.975 (Yeast dataset); ACC = 0.889, F1 = 0.893, MCC = 0.781, AUC = 0.891 (Helicobacter pylori dataset)	Uses PSSM‐based feature representation, CNN for deep feature extraction, and FSRF for noise filtering to improve prediction accuracy; Fast computation, suitable for large‐scale PPI prediction
PIPR^[^ ^93^ ^]^	Siamese Residual RCNN	Yeast dataset, Guo's dataset, STRING dataset, SKEMPI dataset	Sequence‐based PPI prediction, applicable to binary classification, interaction type recognition, and binding affinity estimation	ACC = 0.971, F1 = 0.971, MCC = 0.942, AUPRC = 0.970 (Yeast dataset); MSE = 0.0063, MAE = 0.0548, Corr = 0.873 (SKEMPI dataset)	Eliminates the need for predefined features, reducing data preprocessing effort; Combines local features and sequential information for improved PPI prediction accuracy; Strong generalization ability, applicable to various PPI tasks
DELPHI^[^ ^96^ ^]^	CNN + RNN (Bi‐GRU)	Dset_186, Dset_72, Dset_164, Dset_448, Dset_355	Predicts PPI‐binding sites, improving accuracy in identifying protein‐protein interaction interfaces	ACC = 0.829, AUPRC = 0.337, MCC = 0.272 (Dset_448); ACC = 0.848, AUPRC = 0.326, MCC = 0.278 (Dset_355); ACC = 0.803, AUPRC = 0.319, MCC = 0.235 (Dset_186); ACC = 0.765, AUPRC = 0.332, MCC = 0.209 (Dset_164); ACC = 0.847, AUPRC = 0.237, MCC = 0.189 (Dset_72)	Uses CNN combined with RNN (Bi‐GRU) for feature extraction and incorporates novel features such as HSP, ProtVec1D, and position information to improve accuracy; Outperforms other PPI‐binding site prediction methods by 18.5% (AUPRC) and 27.7% (MCC)
HIGH‐PPI^[^ ^98^ ^]^	Dual‐view Graph Neural Network (BGNN + TGNN)	STRING database (SHS27k subset, containing 1690 proteins and 7624 PPI relationships)	Integrates protein structure and PPI network topology for high‐accuracy PPI prediction and identification of key binding and catalytic sites	AUPR = 0.930 (Binding), AUPR = 0.908 (Reaction), AUPR = 0.835 (Ptmod), AUPR = 0.885 (Catalysis), AUPR = 0.835 (Inhibition)	Uses a dual‐view graph learning strategy, combining internal protein structure information and external network topology for improved prediction accuracy; Strong generalization across different PPI tasks; Provides interpretable PPI insights by identifying key binding sites.
GNN‐PPI^[^ ^99^ ^]^	GCN + GAT	Human PPI dataset (Pan's dataset), S. cerevisiae dataset (DIP)	Predicts PPIs by integrating protein sequence and structural information to improve interaction recognition accuracy	ACC = 0.981, F1 = 0.987, MCC = 0.952, AUPRC = 0.989 (Human dataset); ACC = 0.948, F1 = 0.948, MCC = 0.897, AUPRC = 0.965 (S. cerevisiae dataset)	Combines GCN and GAT to leverage both protein structure and sequence features for improved PPI prediction; Uses pre‐trained protein language models (SeqVec, ProtBert) to enhance node feature representation; Outperforms sequence‐based PPI methods with better generalization ability
EnsemPPIS^[^ ^119^ ^]^	Transformer + Gated CNN	DeepPPISP dataset, DELPHI dataset, GraphPPIS dataset	Predicts PPI‐binding sites using only protein sequences at the residue level	MCC = 0.277, AUPRC = 0.405, F1 = 0.405 (DeepPPISP task); MCC = 0.291, AUPRC = 0.354, F1 = 0.385 (DELPHI task)	Uses Transformer and Gated CNN to extract residue interaction features, combining global and local sequence features via ensemble learning; Outperforms DELPHI by 8.8% (AUPRC), 5.8% (F1), and 4.7% (MCC)
PRoBERTa^[^ ^120^ ^]^	Transformer (BERT‐based)	UniProtKB/Swiss‐Prot (450K protein sequences), HIPPIE database (for PPI prediction)	Pre‐trained Transformer‐based model for protein sequence representation, applied to protein family classification and PPI prediction	Protein Family Classification: ACC = 0.980 (Binary), ACC = 0.920 (Multi‐class); PPI Prediction (Conservative Scenario): ACC = 0.960, Precision = 0.950, Recall = 0.970; PPI Prediction (Aggressive Scenario): ACC = 0.790, Precision = 0.820, Recall = 0.730	Uses RoBERTa‐based Transformer for protein sequence modeling, enabling high‐accuracy prediction without structural information; Outperforms DeepFam and PIPR in PPI prediction, with a 170× speedup in training; Strong generalization ability for multiple protein prediction tasks.
RoseTTAFold^[^ ^121^ ^]^	Three‐Track Neural Network (1D sequence, 2D distance map, 3D coordinates)	PDB database, CASP14 assessment data, CAMEO evaluation data	Predicts the 3D structure of both monomeric proteins and protein‐protein complexes, improving accuracy in structural biology modeling	CASP14 Evaluation: Average lDDT = 0.750; CAMEO Evaluation: Outperforms all servers on 69 protein targets	Integrates 1D, 2D, and 3D representations to optimize sequence‐residue distance and coordinate relationships for improved accuracy; Directly predicts protein‐protein complex structures from sequence without prior monomer modeling and docking
RoseTTAFold All‐Atom^[^ ^109^ ^]^	Three‐Track Neural Network + Molecular Graph Representation	PDB database, CAMEO evaluation data, PoseBusters dataset	Predicts and models full atomic‐level biomolecular complexes containing proteins, nucleic acids, small molecules, metal ions, and covalent modifications	Protein‐Small Molecule Complex Prediction: 43% of predictions with ligand RMSD < 2Å (CAMEO evaluation), 4× higher success rate than AutoDock Vina; Covalent Modification Prediction: 46% of predicted modifications have RMSD < 2.5Å (PDB test set)	Uses molecular graph representation and three‐track architecture to model protein interactions with small molecules, metals, and nucleic acids; Directly predicts full complexes without prior structure information, surpassing existing methods; Integrates RFdiffusionAA for small molecule binder design, experimentally validated
RoseTTAFoldNA^[^ ^105^ ^]^	Three‐Track Neural Network	PDB database (including protein‐nucleic acid complexes), RNAcentral, Rfam, Cis‐BP databases	Predicts the 3D structures of protein‐nucleic acid (protein‐RNA and protein‐DNA) complexes, improving modeling accuracy for protein‐nucleic acid interactions	Protein‐Nucleic Acid Complex Prediction (224 monomeric complexes): lDDT = 0.730, 45% of models have FNAT > 0.5; Multisubunit Protein‐NA Complexes (161 cases): lDDT = 0.720, 30% of complexes with lDDT > 0.8	Uses a three‐track neural network to optimize protein‐nucleic acid interaction predictions, significantly outperforming docking‐based methods (e.g., AlphaFold‐Hdock); Predicts protein‐NA complexes without requiring known templates, achieving high accuracy in 31% of cases without homologs
GeoBind^[^ ^106^ ^]^	Geometric Deep Learning + Quasi‐Geodesic Convolutional Neural Networks	BioLip database (DNA‐195, RNA‐157), GraphBind benchmark dataset	Predicts nucleic acid binding interfaces on protein surfaces, improving protein‐nucleic acid interaction modeling accuracy	DNA binding site prediction (DNA‐195 dataset): AUROC = 0.941, AUPRC = 0.572; RNA binding site prediction (RNA‐157 dataset): AUROC = 0.912, AUPRC = 0.563; GraphBind dataset: DNA binding MCC improved by 5.1%‐50.2%, RNA binding MCC improved by 13.7%‐49.3%	Uses geometric deep learning to directly process protein surface point clouds, enhancing nucleic acid binding site identification; Outperforms GraphBind, DRNApred, and other methods in AUPRC and MCC metrics; Supports multimer complex prediction, achieving better accuracy than monomer‐based predictions
DeepSite^[^ ^107^ ^]^	Bidirectional LSTM (BLSTM) + CNN	ENCODE dataset (690 ChIP‐seq experiments)	Predicts DNA‐protein binding sites, improving the accuracy of DNA‐binding protein recognition	ENCODE dataset test: ACC = 0.892, Sen = 0.871, Spe = 0.911, MCC = 0.783	Uses BLSTM to capture long‐ and short‐range dependencies in DNA sequences, with CNN for feature extraction; Improves accuracy by 6.89%, sensitivity by 5.28%, specificity by 8.35%, and MCC by 0.138 over conventional CNN models; Outperforms DeepBind and DeepSEA methods
PrismNet^[^ ^108^, ^137^ ^]^	CNN + 2D & 1D Residual Network + Attention Mechanism	ENCODE eCLIP dataset (168 RBPs), POSTAR database, icSHAPE RNA structural data (7 cell types: K562, HepG2, HEK293T, HeLa, etc.)	Predicts RBP binding by integrating RNA sequences and in vivo RNA structure, supporting dynamic cell‐specific RBP binding modeling	AUROC = 0.850, AUPRC = 0.764;	First deep learning model integrating in vivo RNA structure data for RBP binding prediction, capturing cell type‐specific RBP binding patterns; Excels in riboSNitch (structural variation site) identification, linking RNA structure changes to human diseases.
LigandMPNN^[^ ^111^ ^]^	Protein‐Ligand Graph Neural Network	PDB database (including protein structures with small molecules, metals, nucleotides), CAMEO evaluation data	Designs protein‐small molecule binding interfaces, improving protein‐ligand affinity and specificity	; Sequence recovery for residues interacting with small molecules(63.3%), nucleotides(50.5%) and metals(77.5%)	Uses protein‐ligand graph neural network to model atomic‐level protein‐small molecule interactions; Improves small molecule binding site prediction by 16.2% over Rosetta and ProteinMPNN; Applicable to enzyme design, receptor‐ligand optimization, and protein‐drug interaction modeling
MaSIF‐neosurf^[^ ^122^ ^]^	Geometric Deep Learning + Molecular Surface Learning	PDB database (14 protein‐small molecule complexes), Experimental validation data (Bcl2‐venetoclax, DB3‐progesterone, PDF1‐actinonin)	Predicts and optimizes protein‐small molecule binding interfaces, enabling the design of novel drug‐binding proteins	Protein‐small molecule complex prediction (14 complexes, 28 test cases): Correct binding site prediction rate 70% (MaSIF‐neosurf) vs. 14% (RoseTTAFold All‐Atom); Binding energy optimization (ΔΔG improved by 17.0%‐27.7%)	Uses MaSIF surface fingerprinting to learn protein‐small molecule binding interfaces, achieving a 5‐fold improvement in binding interface recognition compared to RoseTTAFold All‐Atom; Enables drug‐induced protein‐protein interaction (PPI) prediction and design, applicable to molecular glues, drug‐target optimization, and small‐molecule‐mediated protein assembly
BiteNet_N_^[^ ^112^ ^]^	3D CNN	PDB database (∼2000 nucleic acid‐small molecule complexes, ∼2500 binding sites)	Predicts nucleic acid‐small molecule binding sites, supporting RNA and DNA binding site recognition and improving nucleic acid‐targeted drug discovery	Nucleic acid‐small molecule binding site prediction (10‐fold cross‐validation on test set): Weighted AP = 0.75, ROC AUC = 0.89, MCC = 0.59; Outperforms RNAsite, RBind, and other methods, improving AUC by ∼15%	Uses voxel‐based 3D representation, combined with 3D CNN for RNA and DNA binding site prediction; More accurate than empirical rule‐based or shallow machine learning approaches; Supports conformation‐specific nucleic acid binding prediction (validated on HIV‐1 TAR RNA, ATP‐aptamer cases)
MultiModRLBP^[^ ^113^ ^]^	Relational Graph Convolutional Network (RGCN) + CNN + Pretrained Model (RNABert)	RNAglib dataset (851 RNA‐small molecule interactions), RLBind training set, Test18 & Test3 datasets	Predicts RNA‐small molecule binding sites, enhancing RNA‐targeted drug development and improving binding specificity	RNA‐small molecule binding site prediction: AUC = 0.780 (Test18), AUC = 0.843 (Test3), MCC improvement of 16.7% (Test18)	Integrates RNA structural information (RGCN), sequence semantics (RNABert), and local/global features (CNN)for prediction; Outperforms RLBind by 5.4% (Test18) and 7.7% (Test3) in AUC; Supports binding site prediction under RNA structural flexibility, applicable for RNA drug target optimization.

## 4 Limitations and Future of AI‐Based Biomolecular Interaction Prediction

Deep learning models have shown remarkable potential in predicting biomolecular interactions. However, they still face substantial challenges in generalizing to unseen test data or real‐world conditions. For protein‐protein and protein‐nucleic acid interactions, the chemical space is relatively constrained, involving only 20 amino acids and 4 nucleic acid bases. In these domains, models like AlphaFold have approached near‐experimental accuracy. In contrast, when dealing with protein‐ligand systems, the nearly infinite chemical space of potential ligands poses a far more complex scenario. Existing training datasets are often too sparse and biased to cover the broad chemical subspaces encountered in real applications. As a result, models may fail to accurately predict binding conformations for novel, chemically distinct ligands and may over‐rely on frequently observed binding patterns in the training data, ultimately limiting their capacity to capture and extrapolate to new chemical structures. Moreover, data leakage refers to the close relationship between training and test datasets, which may result in an overestimation of model performance and reduced generalizability to new datasets. This issue is particularly prevalent in biological studies, such as those involving protein sequences and structures, where training and test data points may be related, for instance, by belonging to the same protein family or having homology.^[^ ^123^ ^]^ Simply using a sequence identity threshold (e.g., 30% or 25%) to distinguish training from test sets is inadequate for preventing data leakage, as homologous proteins can exhibit low sequence similarity yet retain the same function.^[^ ^124^, ^125^ ^]^ To mitigate data shortage, more sensitive tools, such as Hidden Markov Model tools (e.g., HH‐suite) or structural classification methods (e.g., CATH or ECOD), should be employed to better identify and eliminate such similarities.^[^ ^126^, ^127^, ^128^ ^]^ Furthermore, data scarcity is particularly pronounced in the context of RNA structural data. While a limited number of high‐resolution protein structures is available, the scarcity is even more severe for RNA, with RNA structures accounting for only ≈3% of the experimentally determined structures in the PDB.^[^ ^129^ ^]^ Although AlphaFold 3 has made significant progress in protein structure prediction, the error rate remains relatively high in nucleic acid structure prediction.

Merely increasing the amount of data does not fully address these issues, as data quality and diversity are also critical factors. Biological datasets are frequently afflicted by experimental noise and measurement biases that can mislead models into learning irrelevant or erroneous features. This problem is exacerbated for rare labels or underrepresented phenomena, where the limited data available is further compromised by noise. Together, these factors severely limit a model's accuracy and transferability. To overcome these constraints, future efforts must focus not only on expanding data volume but also on enhancing data quality, diversity, and representativeness, as well as on employing more robust model architectures and training strategies that reduce overfitting to noisy signals.^[^ ^123^, ^130^ ^]^

Interpretability refers to the ability to understand, explain, and trace the reasoning behind a model's decisions.^[^ ^123^ ^]^ Deep learning models are often regarded as “black boxes” owing to their complex network structures and nonlinear characteristics, which render it challenging for humans to comprehend how specific predictions or conclusions are reached. The interpretability of a model is dependent on the machine learning method employed and the input data. Compared to neural networks, non‐neural network models are generally more interpretable, as these methods typically utilize intuitive feature sets and fewer parameters. However, for neural networks, particularly deep neural networks, the explanation of model behavior becomes more challenging, as they typically involve a large number of input features and parameters. For example, saliency maps can be utilized to identify regions of an input image that are most significant for a specific classification, although determining precisely how the attributes of the data influence the prediction remains challenging. These saliency maps can serve as a “sanity check” to ensure that the model focuses on task‐relevant parts of the data rather than relying on unrelated information (e.g., labels within the image).^[^ ^131^ ^]^

The biophysical nature of biomolecular interactions cannot be overlooked. Most contemporary predictive models for biomolecular interactions are designed to identify potential binding pairs between target molecules and their ligands. These approaches often rely on databases containing known interaction pairs, structural similarities, or sequence homology to facilitate predictions. However, these models frequently overlook the complex dynamics underlying biomolecular interactions. For instance, current AI methods may match a small molecule to a protein pocket based on geometric compatibility and historical interaction records but fail to account for the subtleties of molecular behavior during the interaction process, such as van der Waals forces, electrostatic interactions, hydrogen bonds, hydrophobic effects, and steric hindrance. This can result in inaccurate predictions, as the myriad forces and interactions involved are not adequately considered. Atomic interactions are governed by intricate physical forces and influenced by the surrounding environment. These combined effects determine the binding sites and affinities of proteins, nucleic acids, and ligands during the docking process. Ignoring these factors that govern biomolecular interactions may result in models that, while seemingly plausible on paper, do not accurately reflect the real‐world behavior of biomolecules. If the AI model is trained on a dataset lacking examples of certain types of interactions (e.g., specific hydrogen bonding networks or particular electrostatic configurations), it may incorrectly predict high affinities for ligands that would not effectively bind in practice. Conversely, docking simulations may require more time but provide a more mechanistic understanding of why certain ligands are favored over others.

In the case of drug discovery, traditional structure‐based drug design involves molecular docking simulations to predict how a ligand fits into a protein's active site. Modern AI models may utilize large datasets to predict binding affinities without explicitly simulating these interactions. Although AI models can be faster and capable of handling larger datasets, they risk missing critical details captured by docking simulations. To enhance prediction accuracy, future models should integrate knowledge of biomolecular interactions into their frameworks. Techniques such as molecular dynamics (MD) simulations combined with deep learning, molecular mechanisms, and features that include interaction fingerprints could be employed. By doing so, these advanced AI models could more effectively capture the nuanced behavior of biomolecules, thereby leading to more reliable predictions.

Feature engineering that incorporates physical properties is crucial for the integration of intermolecular forces. By utilizing quantum chemistry, classical force fields, or MD simulations, specific intermolecular force values—such as hydrogen bond count, van der Waals energy, and charge distribution—can be obtained.^[^ ^132^, ^133^, ^134^ ^]^ These numerical features can be encoded and utilized as inputs for neural networks, thereby guiding them to learn the microscopic mechanisms of molecular interactions. Electrostatic potential fields and hydrophobicity distributions, for instance, can be represented as feature matrices combined with molecular structural information, thereby enhancing the model's ability to predict binding sites and affinities with greater accuracy. Physics‐Informed Neural Networks (PINNs) represent an advanced approach that embeds physical equations directly into the neural network's loss function, thereby ensuring compliance with physical laws while fitting data.^[^ ^135^ ^]^ For example, in protein‐ligand docking, Physics‐Informed Neural Networks may utilize formulas related to binding free energy—including van der Waals energy, electrostatic energy, and entropy contributions—to guide the neural network, thereby making its predicted binding modes more consistent with physical principles.

Many AI models focus primarily on the structural compatibility between drug molecules and their target receptors. Although this approach can yield useful predictions, it fails to account for the dynamic nature of molecular interactions. In reality, the binding process is influenced by various environmental conditions, such as ion concentration, pH levels, and temperature. These factors can significantly influence the strength and specificity of the interaction.

Ignoring these environmental variables may lead to inaccurate predictions and misguided therapeutic strategies.^[^ ^136^ ^]^ For example, if an AI model predicts a strong binding affinity between a drug and its receptor without accounting for the high salt concentration in the cellular environment, the actual effectiveness of the drug could be overestimated. This occurs because an increased ion concentration can screen charges on the protein surface, thereby reducing electrostatic interactions and weakening the binding affinity.

With an increasing number of research examples and real‐world applications, the impact of AI on predicting biomolecular interactions has become increasingly profound. Advances in AI have enabled us to analyze complex biomolecular interactions with unprecedented precision and efficiency, providing significant momentum for drug discovery, disease research, and personalized medicine. From protein‐protein interactions to protein‐small molecule and protein‐nucleic acid bindings, AI technology is revolutionizing traditional experimental methods, delivering faster and more accurate predictions. As algorithms continue to improve and training datasets expand, AI is expected to play an even greater role in predicting more complex biomolecular systems in the future. Particularly in clinical applications, AI will provide more effective tools for targeted therapies, vaccine development, and new drug design. It is foreseeable that AI will become an indispensable core technology in modern biomedical research, driving medical innovation to new heights.

## Conflict of Interest

The author declares no conflict of interest.
